# Sinapyl Alcohol Derivatives from the Lipo-soluble Part of *Dichrocephala benthamii* C. B. Clarke

**DOI:** 10.3390/molecules18021720

**Published:** 2013-01-29

**Authors:** Xinhui Tian, Gang Ding, Chaozhong Peng, Yanbao Hu, Li Li, Hong Chen, Zhongmei Zou

**Affiliations:** 1 Key Laboratory of Bioactive Substances and Resources Utilization of Chinese Herbal Medicine, Ministry of Education, Institute of Medicinal Plant Development, Chinese Academy of Medical Sciences and Peking Union Medical College, Beijing 100193, China; 2 State Key Laboratory of Bioactive Substance and Function of Natural Medicines, Institute of Materia Medica, Chinese Academy of Medical Sciences and Peking Union Medical College, Beijing 100050, China; 3 Tianjin Key Laboratory for Biomarkers of Occupational and Environmental Hazard, Department of Pharmacognosy, Logistics University of Chinese People’s Armed Police Forces, Tianjin 300162, China

**Keywords:** *Dichrocephala benthamii* C. B. Clarke, Asteraceae, sinapyl alcohol derivatives, absolute configuration determination, cytotoxicity

## Abstract

Four new sinapyl alcohol derivatives dichrocephols A–D (compounds **1**–**4**) were isolated from the lipo-soluble part of the whole herb of *Dichrocephala benthamii* C. B. Clarke, together with the known compound syringenin isovalerate (**5**). Their structures were elucidated on the basis of spectroscopic analysis. Their absolute configurations were established by the method of alkaline hydrohysis. Compounds **1**–**3** showed moderate cytotoxity against HeLa cells, with IC_50_ values of 14.8 μM, 51.6 μM and 81.6 μM, respectively. This is the first time that sinapyl alcohol derivatives were isolated from the genus *Dichrocephala*.

## 1. Introduction

The Asteraceae plant *Dichrocephala benthamii* C. B. Clarke is an annual herb which is only distributed in China and India [1]. Its whole herb has commonly been used as a fork medicine among the Dai nationality of China for the treatment of indigestion, common cold and fever in children, pneumonia and hepatitis [2,3]. However, to the best of our knowledge, there have been few previous reports on phytochemical investigations of this species [4]. In our search for new bioactive constituents from the medicinal plants used by the Dai nationality in China, we initiated chemical studies of the whole herb of *D. benthamii*.

**Figure 1:**
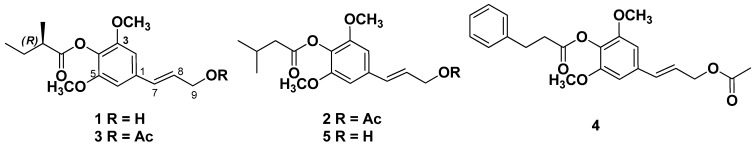
Structures of compounds **1**–**5**.

The air-dried and chopped whole herbs of *D. benthamii* were extracted with 95% EtOH. After removal of solvent, the residue was subject to the diatomaceous silica and bathed successively with petroleum ether, CH_2_Cl_2_, EtOAc and MeOH. The petroleum ether part led to the isolation of four new sinapyl alcohol derivatives **1**–**4** and the known compound syringenin isovalerate (**5**) (Figure 1) [5].

## 2. Results and Discussion

Compound **1**, a colorless oil, had the molecular formula of C_16_H_22_O_5_, as determined by analysis of its HRESIMS (*m/z* 317.1358 [M+Na]^+^). The IR spectrum of **1** showed absorption bands at 1757 (C=O), 1596, 1507, 1463 (-phenyl) cm^−1^. Analysis of the ^1^H-, ^13^C-, DEPT, and HSQC NMR data revealed that there was a 1,3,4,5-tetrasubstituted phenyl in **1**. The signals at *δ*_C_ 56.6 and *δ*_H_ 3.84 (6H, s) indicated that there were two overlapped methoxy groups, which were symmetrically located on the phenyl ring. Interpretation of the ^1^H-^1^H COSY NMR data of **1** led to the identification of two isolated proton spin-systems corresponding to C-7−C-9, C-2′−C-5′ units. The remaining fragment connections were determined by HMBC data, and established a skeleton similar to that of sinapyl alcohols [5,6]. The HMBC correlations from *δ*_H_ 6.79 (H-2) to *δ*_C_ 137.2 (C-1), 131.4 (C-7), *δ*_H_ 6.61 (H-7) to *δ*_C_ 137.2 (C-1), 104.3 (C-2, 6), *δ*_H_ 6.39 (H-8) to *δ*_C_ 137.2 (C-1) indicated that C-1 was connected to C-7. The HMBC correlations from *δ*_H_ 1.64 (1H, m, H-3′a), 1.80 (1H, m, H-3′b), 1.30 (3H, d, *J* = 7.2 Hz, H-5′) to *δ*_C_ 176.5 (C-1′) indicated that there was a 2-methylbutyryl group in **1**. Due to absence of HMBC correlation between *δ*_H_ 4.27 (H-9) and *δ*_C_ 176.5 (C-1′) together with consideration of the chemical shift values of C-9, and compared with its analogues [5,6], 2-methylbutyryloxy was suggested to be connected with C-4. Thus the planar structure of **1** was established as (*E*)-3,5-dimethoxy-4-(2-methylbutyryloxy) phenylpropanol.

The absolute configuration at C-4 in the 2-methylbutyryl group was established by the alkaline hydrolysis method. According to the *α*_D_ values of (−14.3) for (*R*)-2-methylbutyric acid and (+19.3) for (*S*)-2-methylbutyric acid and compared with the data reported in the literature [7,8,9,10,11], the absolute configuration of the 2-methylbutyryl group in **1** was supposed as *R* by the negative α_D_ value {[α${]}_{D}^{25}$ −4.6 (c 0.04, MeOH)} of the 2-methylbutyric acid. Therefore the structure of **1** was established as (*E*)-3, 5-dimethoxy-4-(2*R*-methylbutyryloxy)phenylpropanol, which was named dichrocephol A.

**Figure 2:**
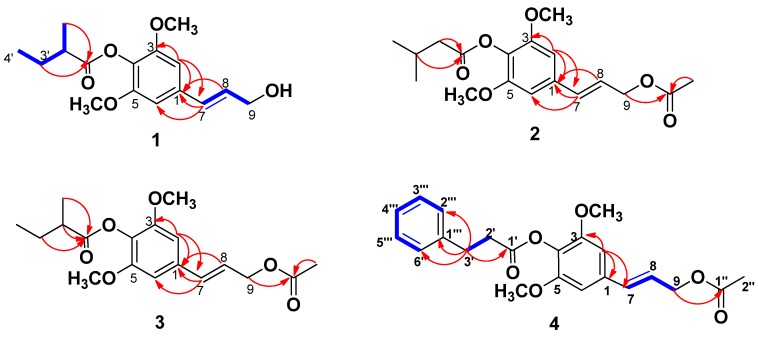
^1^H–^1^H COSY, HMBC for compounds **1**–**4**.

Compound **2**, a colorless oil, was assigned the molecular formula C_18_H_24_O_6_ by HRESIMS analysis (*m/z* 359.1510 [M+Na]^+^). The ^1^H- and ^13^C-NMR data of **2** was similar to that of **1** except the substituent at C-4 and C-9. The signals at *δ*_H_ 2.04 (3H, s, H-2′′) and *δ*_C_ 170.9 (C-1′′), 20.8 (C-2′′) indicated the existence of the acetyl group. In addition, the signals at *δ*_H_ 1.06 (6H, d, *J* = 6.6 Hz, H-4′, 5′), 2.24 (1H, m, *J* = 7.2, 6.6 Hz, H-3′), 2.41 (2H, d, *J* = 7.2 Hz, H-2′) together with the HMBC correlations shown in Figure 2 revealed that isopentanoyl had replaced the 2-methylbutyryl group in **1**. The overlapped signals of C-1′′and C-1′ in ^13^C-NMR led to the complexity that the two substituents could not be determined to connect with C-4 or C-9 by HMBC correlations. Detailed analysis of the chemical shift values of **2**, and comparison of its analogues implied that isopentanoyloxy should be anchored at C-4 [5,6]. The EIMS fragment ions of **2** also supported the above-mentioned deduction. 

**Figure 3:**
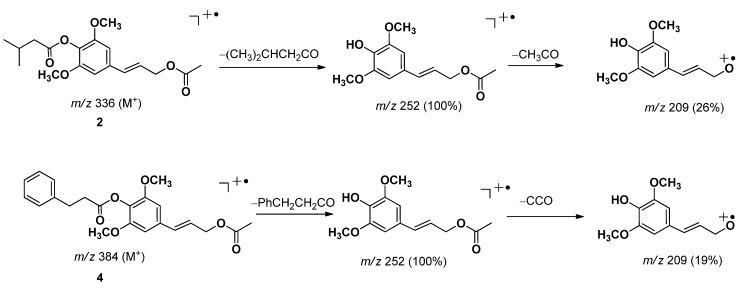
EIMS fragment ions of **2** and **4**.

The abundance the peak of *m/z* 252 was 100%, which implied that the isopentanoyl moiety was repelled by the two methoxy units and lost easily (Figure 3). Therefore 2 was established as (*E*)-3,5-dimethoxy-4-isopentanoyloxy phenylpropanol acetate, which was named dichrocephol B.

Compound **3**, a colorless oil, was isolated together with **2**. The mole ratio of **3** to **2** was determined to be 1:0.6 according to their ^1^H-NMR integration. The ^1^H and ^13^C-NMR data of **3** was very similar to that of **2**, except that isopentanoyl in **2** was replaced by the 2-methylbutyl group, which was confirmed by HMBC correlations. The absolute configuration at C-4 in the 2-methylbutyl group was also established by alkaline hydrolysis method, and was suggested as *R* by the negative α_D_ value {[α${]}_{D}^{25}$ −8.0 (c 0.05, MeOH)} of the 2-methylbutyric acid. Thus the structure of **3** was established as (*E*)-3, 5-dimethoxy-4-(2*R*-methylbutyryloxy)phenylpropanol acelate, which was named dichrocephol C.

Compound **4**, a colorless oil, had the molecular formula C_22_H_24_O_6_, as determined by analysis of its HRESIMS (*m/z* 385.1640 [M+H]^+^). Analysis of the ^1^H and ^13^C-NMR data revealed the same structural of (*E*)-3, 5-dimethoxyphenylpropanol acelate as those in compound **2** and **3**. The ^1^H-NMR signals at *δ*_H_ 2.90 (2H, t, *J* = 6.5 Hz, H-2′), 3.05 (2H, t, *J* = 6.5 Hz, H-3′), 7.23 (1H, m, *J* = 7.0, 1.5 Hz, H-4′′′), 7.36 (4H, m, H-2′′′, 3′′′, 5′′′, 6′′′), and the HMBC correlations from *δ*_H_ 3.05 (H-3′) to *δ*_C_ 170.8 (C-1′), 141.7 (C-1′′′), 129.3 (C-2′′′, 6′′′) both indicated the existence of the phenylpropionyl group [12,13]. The HMBC correlations from *δ*_H_ 4.71 (H-9) to *δ*_C_ 170.8 (C-1′′ and C-1′ were overlapped) could not determine that the phenylpropionyloxy and acetyloxy groups was connected with C-4 or C-9, whereas taking account for the chemical shift values and compared with NMR data of compounds **1**–**3**, the phenylpropionyloxy group was supposed to connect to C-4. This hypothesis was also supported by the EIMS spectra the same way as those of **2** (Figure 3). Therefore, compound **4** was characterized as (*E*)-3, 5-dimethoxy-4-phenylpropionyloxyphenylpropanol acetate, which was named dichrocephol D. 

The sinapyl alcohol derivatives exhibited notable cytotoxicity against KB, BEL-7404 cancer cell lines [14] and HIV-1 replication [15]. Therefore, compounds **1**–**5** were tested for their cytotoxicity against HeLa cell lines [16] and inhibitory effect against COX-2 [17] by applying MTT and initial velocity method respectively. The results showed that compounds **1**–**3** showed moderate cytotoxic activities against HeLa cells with IC_50_ values of 14.8 μM, 51.6 μM, and 81.6 μM respectively. All of these compounds had no significant effect against COX-2 enzyme unfortunately.

## 3. Experimental

### 3.1. General

Optical rotations were measured on a Perkin-Elmer 241 polarimeter (Waltham, MA, USA), and UV data were recorded on Beckman Coulter DU 800 spectrometer (Tokyo, Japan). IR data were recorded using a Shimadzu FTIR-8400S spectrophotometer (Tokyo, Japan). ^1^H and ^13^C-NMR data were acquired with a Bruker 600 (Munich, Germany) and Bruker 500 instruments using the solvent signals (CD_3_OD: *δ*_H_ 3.30/*δ*_C_ 49.0, CD_3_COCD_3_: *δ*_H_ 2.05/*δ*_C_ 29.9, 206.7) as references. HRESIMS data were acquired using a LTQ Orbitrap XL mass spectrometer (Santa Clara, CA, USA). EIMS data were recorded on a GCMS-QP 2010 Shimadzu spectrometer (Tokyo, Japan). Sephadex LH-20 (Pharmacia Biotech AB, Uppsala, Sweden), diatomaceous silica, silica gel (80–100, 100–200, 200–300 mesh) and silica gel GF254 sheets (0.20–0.25 mm) (Qingdao Marine Chemical Plant, Qingdao, China) were used for column chromatography and TLC, respectively.

### 3.2. Plant Material

The whole herb of *D. benthamii* was collected from Xishuangbanna county, Yunnan Province of People’s Republic of China in October 2008. The sample was identified by one of the authors C. Z. Peng and the voucher specimen (No. DB2008) has been deposited in the Herbarium of the Institute of Medicinal Plant Development, Chinese Academy of Medical Sciences, Beijing.

### 3.3. Extraction and Isolation

The air-dried and chopped whole herbs of *D. benthamii* (20 kg) were extracted with 95% EtOH (3 × 80 L) at 70 °C for 1 h/two times. The 95% EtOH extracts were concentrated under reduced pressure at 50 °C. The residue (1.7 kg) was subject to the diatomaceous silica (45 × 18 cm, 1.8 kg) and bathed successively with petroleum ether, CH_2_Cl_2_, EtOAc and MeOH. The petroleum ether part (275 g) was subject to silica gel column chromatography (CC) (45 × 12 cm, 80–100 mesh, 2.0 kg), using petroleum ether–Me_2_CO gradient elution (1:0–0:1) to afford 6 corresponding fractions (A–F). Fraction C (43 g) was fractionated over silica gel CC (50 × 12.5 cm, 100–200 mesh, 1.0 kg) using petroleum ether-Me_2_CO elution (30:1) to give seven fractions (C-1–C-7). The mixture of **2** and **3** (290 mg) was isolated from fraction C-4 (4.4 g) by further chromatography over silica gel CC (60 × 4.5 cm, 200–300 mesh, 220 g), using petroleum ether-Me_2_CO (30:1) elution. Fraction C-5 (1.6 g) was further fractionated by silica gel CC (50 × 3.5 cm, 200–300 mesh, 80 g) using petroleum ether-Me_2_CO (10:1) elution to give five fractions (C-5a–C-5e). Fraction C-5c (50 mg) was purified by semipreparative HPLC (70% aqueous MeOH, 2 mL/min, 210 nm) to afford **2** (*t*_R_ 34.4 min; 33.0 mg). Fraction C-5d (100 mg) was purified by semipreparative HPLC (80% aqueous MeOH, 2 mL/min, 210 nm) to afford **4** (*t*_R_ 20.0 min; 9.0 mg). Fraction E (26 g) was fractionated by silica gel CC (50 × 3.5 cm, 100–200 mesh, 0.5 kg) using petroleum ether-Me_2_CO elution (5:1) to give four fractions (E-1–E-4). Fraction E-4 (3.8 g) was fractionated over silica gel CC (60 × 4.0 cm, 200–300 mesh, 200 g) using petroleum ether-Me_2_CO elution (4:1) to give three fractions (E-4a–E-4c). Fraction E-4b (150 mg) was purified by semipreparative HPLC (65% aqueous MeOH, 2 mL/min, 210 nm) to afford **5** (*t*_R_ 27.8 min; 4.9 mg) and **1** (*t*_R_ 29.2 min; 17.9 mg).

### 3.4. Spectral Data

*Dichrocephol A* (**1**): colorless oil; [α${]}_{D}^{25}$ −2.55 (c 0.19, MeOH); UV (MeOH) λ_max_ (log *ε*) 219 (2.4); IR (KBr) *ν*_max_ 2967, 1757, 1596, 1507, 1463, 1244, 1147, 845 cm^−1^; EIMS *m/z* (%): 294 [M]^+^ (3), 210 (88), 182 (28), 167 (23), 77 (13), 57 (100); HRESIMS *m/z*: 317.1358 [M+Na]^+^ (calcd for C_16_H_22_O_5_Na, 317.1365); ^1^H-NMR (600 MHz, CD_3_OD) and ^13^C-NMR (150 MHz, CD_3_OD) data see Table 1.

*Dichrocephol B* (**2**): colorless oil; UV (MeOH) λ_max_ (log *ε*) 220 (2.6); IR (KBr) *ν*_max_ 2959, 1738, 1597, 1507, 1456, 1245, 1132 cm^−1^; EIMS *m/z* (%): 336 [M]^+^ (3), 252 (100), 209 (26), 149 (8), 57 (41); HRESIMS *m/z* 359.1510 [M+Na]^+^ (calcd for C_18_H_24_O_6_Na, 359.1471); ^1^H-NMR (600 MHz, CD_3_COCD_3_) and ^13^C-NMR (150 MHz, CD_3_COCD_3_) see Table 1.

*Dichrocephol C* (**3**): colorless oil; [α${]}_{D}^{25}$ −4.80 (c 0.06, acetone); UV (acetone) λ_max_ (log *ε*) 230 (3.7); IR (KBr) *ν*_max_ 2964, 1732, 1597, 1244, 1130, 848 cm^−1^; EIMS *m/z* (%): 336 [M]^+^ (3), 252 (100), 209 (26), 149 (8), 105 (5), 77 (5), 57 (41); HRESIMS *m/z* 359.1510 [M + Na]^+^ (calcd for C_18_H_24_O_6_Na, 359.1471); ^1^H-NMR (600 MHz, CD_3_COCD_3_) and ^13^C-NMR (150 MHz, CD_3_COCD_3_) data see Table 1.

*Dichrocephol D* (**4**): colorless oil; UV (MeOH) λ_max_ (log *ε*) 218 (2.4); IR (KBr) *ν*_max_ 2936, 1738, 1597, 1506, 1455, 1245, 1131, 965, 847 cm^−1^; EIMS *m/z* (%): 384 [M]^+^ (4), 327 (8), 267 (13), 252 (100), 209 (19), 134 (41), 105 (61), 91 (85), 77 (35), 55 (100); HRESIMS *m/z*: 385.1640 [M+H]^+^ (calcd for C_22_H_25_O_6_, 385.1651); ^1^H-NMR (500 MHz, CD_3_COCD_3_) and ^13^C-NMR (125 MHz, CD_3_COCD_3_) see Table 1.

### 3.5. Absolute Configurations at C-4 in the 2-Methylbutyryl Group of Compounds **1** and **3**

A mixture of **2** and **3** (20.0 mg) was dissolved in EtOH (2.0 mL) and treated with 5% KOH in H_2_O (4.0 mL). After stirring at room temperature for 24 h, the reaction mixture was concentrated and then partitioned between EtOAc and H_2_O. After extraction with EtOAc three times, the aqueous layer was acidified with HCl to pH = 3.0 and then extracted with CH_2_Cl_2_ three times. The CH_2_Cl_2_ layer was combined and subjected to Sephadex LH-20 CC (CH_2_Cl_2_–MeOH, 1:1) to yield a mixture of 2-methybutyric acid and isopentoic acid (1.5 mg). Since isopentoic acid is optically inactive, the absolute configuration at C-4 of 2-methybutyric acid was identified as *R* by the α_D_ value of {[α${]}_{D}^{25}$ −8.0 (c 0.05, MeOH)}. In the same way, the absolute configuration of C-4 in the 2-methylbutyryl group of **1** was also established as *R* by its α_D_ value of {[α${]}_{D}^{25}$ −4.6 (c 0.04, MeOH)}.

### 3.6. Bioassays

The cytotoxic activities of the isolated compounds were evaluated against HeLa cell line by MTT colorimetric method with 5-fluorouracil as positive control (IC_50_ value 5.9 μM). The inhibitory effect of compounds **1**−**5** on COX-2 (sheep) enzyme (Gayman 760111) were tested by initial velocity method.

## 4. Conclusions

Four new sinapyl alcohol derivatives dichrocephols A–D **1**–**4** were isolated from the lipo-soluble part of the whole herb of *Dichrocephala benthamii* C. B. Clarke, together with the known syringenin isovalerate (**5**). Compounds **1**–**3** showed moderate cytotoxities against HeLa cells with IC_50_ values of 14.8 μM, 51.6 μM and 81.6 μM respectively. This is the first time that sinapyl alcohol derivatives were isolated from the genus *Dichrocephala*.

## Figures and Tables

**Table 1:** ^1^H and ^13^C-NMR Spectroscopic Data for dichrocephols A–D (**1**–**4**).

Pos	1 ^a^ (J in Hz)		2 ^b^ (J in Hz)		3 ^b^ (J in Hz)		4 ^b^ (J in Hz)	
	δ_C_	δ_H_	δ_C_	δ_H_	δ_C_	δ_H_	δ_C_	δ_H_
1	137.2 C		135.6 C		134.6 C		135.8 C	
2	104.3 CH	6.79 s	104.2 CH	6.85 s	103.5 CH	6.62 s	104.3 CH	6.87 s
3	153.7 C		153.4 C		152.4 C		153.5 C	
4	130.7 C		135.6 C		134.6 C		135.8 C	
5	153.8 C		153.4 C		152.4 C		153.5 C	
6	104.3 CH	6.79 s	104.2 CH	6.85 s	103.5 CH	6.62 s	104.3 CH	6.87 s
7	131.4 CH	6.61 d (16.2)	134.1 CH	6.65 d (16.2)	134.2 CH	6.57 d (15.6)	134.2 CH	6.68 d (16.0)
8	130.7 CH	6.39 dt (16.2, 5.4)	125.0 CH	6.36 dt (16.2, 6.6)	123.6 CH	6.20 dt (15.6, 6.6)	125.1 CH	6.39 dt (16.0, 6.5)
9	63.6 CH_2_	4.27 dd (5.4, 1.8)	65.2 CH_2_	4.70 dd (6.0, 0.6)	65.0 CH_2_	4.71 dd (6.6, 0.6)	65.2 CH_2_	4.71 dd (6.5, 1.5)
OCH_3_	56.6 CH_3_	3.84 s	56.4 CH_3_	3.83 s	56.2 CH_3_	3.81 s	56.6 CH_3_	3.83 s
1′	176.5 C		170.9 C		174.5 C		170.8 C	
2′	42.4 CH	2.66 m	43.4 CH_2_	2.41 d (7.2)	41.1 CH	2.68 m	35.8 CH_2_	2.90 t (6.5)
3′	28.1 CH_2_	1.64 m, 1.80 m	26.7 CH	2.24 m	27.1 CH_2_	1.61 m, 1.83 m	31.6 CH_2_	3.05 t (6.5)
4′	11.9 CH_3_	1.07 t (7.2)	22.5 CH_3_	1.06 d (6.6)	11.6 CH_3_	1.03 t (7.8, 7.2)		
5′	17.4 CH_3_	1.30 d (7.2)	22.5 CH_3_	1.06 d (6.6)	16.9 CH_3_	1.29 d (6.6)		
1′′			170.9 C		170.9 C		170.8 C	
2′′			20.8 CH_3_	2.04 s	21.1 CH_3_	2.10 s	20.9 CH_3_	2.04 s
1′′′							141.7 C	
2′′′-6′′′							129.3 C	7.36 m
4′′′							127.1 C	7.23 tt (7.0, 1.5)

*^a^* recorded in CD_3_OD; *^b^* recorded in CD_3_COCD_3_.

## Supplementary Materials

Supplementary materials can be accessed at: http://www.mdpi.com/1420-3049/18/2/1720/s1.
