# Supplementary material for: Sinapyl Alcohol Derivatives from the Lipo-soluble Part of Dichrocephala benthamii C. B. Clarke
Source: Molecules. 2013 Jan 29;18(2):1720–7. doi: 10.3390/molecules18021720 (PMC6269932; doi:10.3390/molecules18021720)

Supporting Information

Figure S1. IR Spectrum of Dichrocephol **A** (**1**).

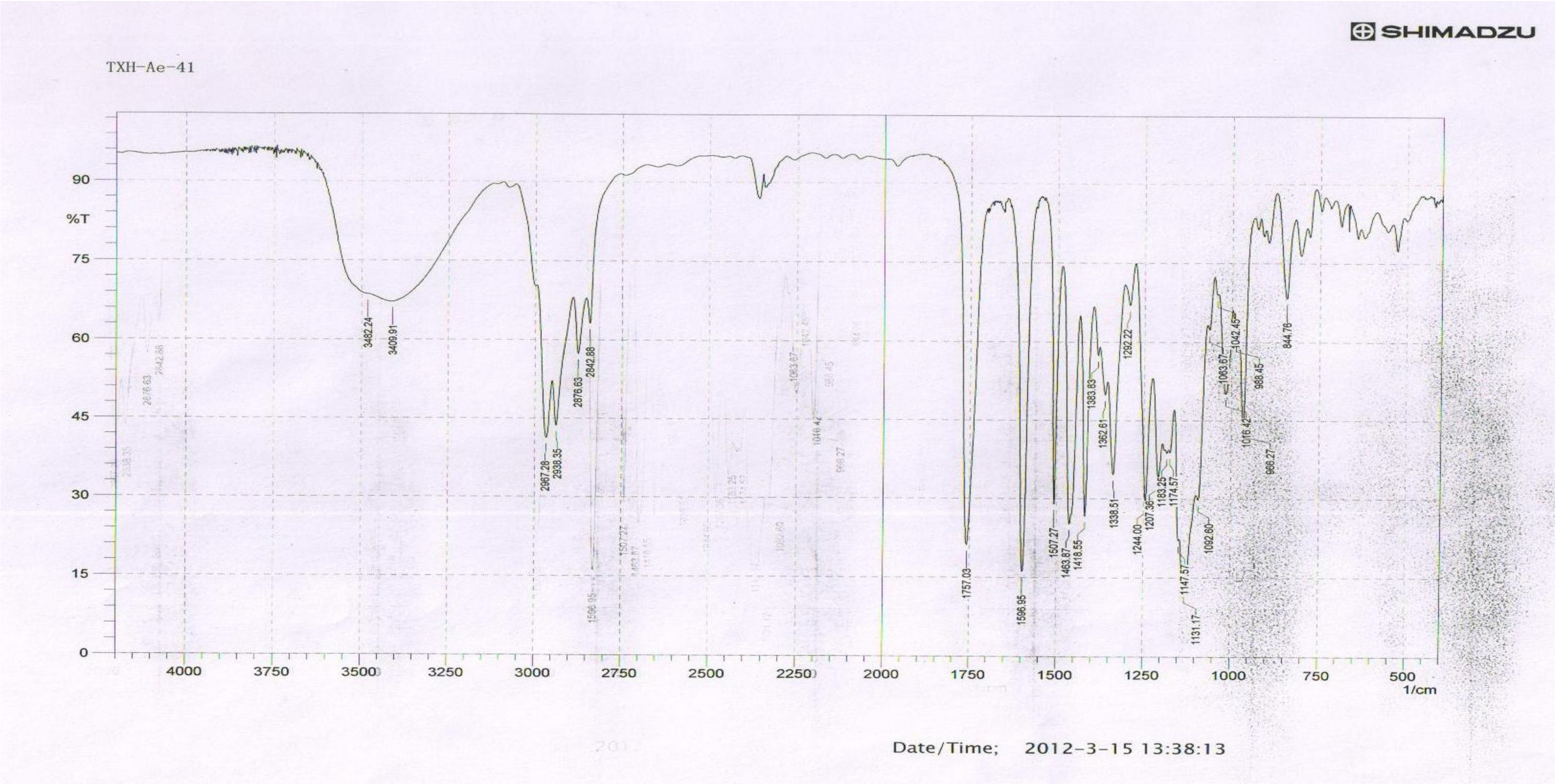

Figure S2. EIMS Spectrum of Dichrocephol **A** (**1**).

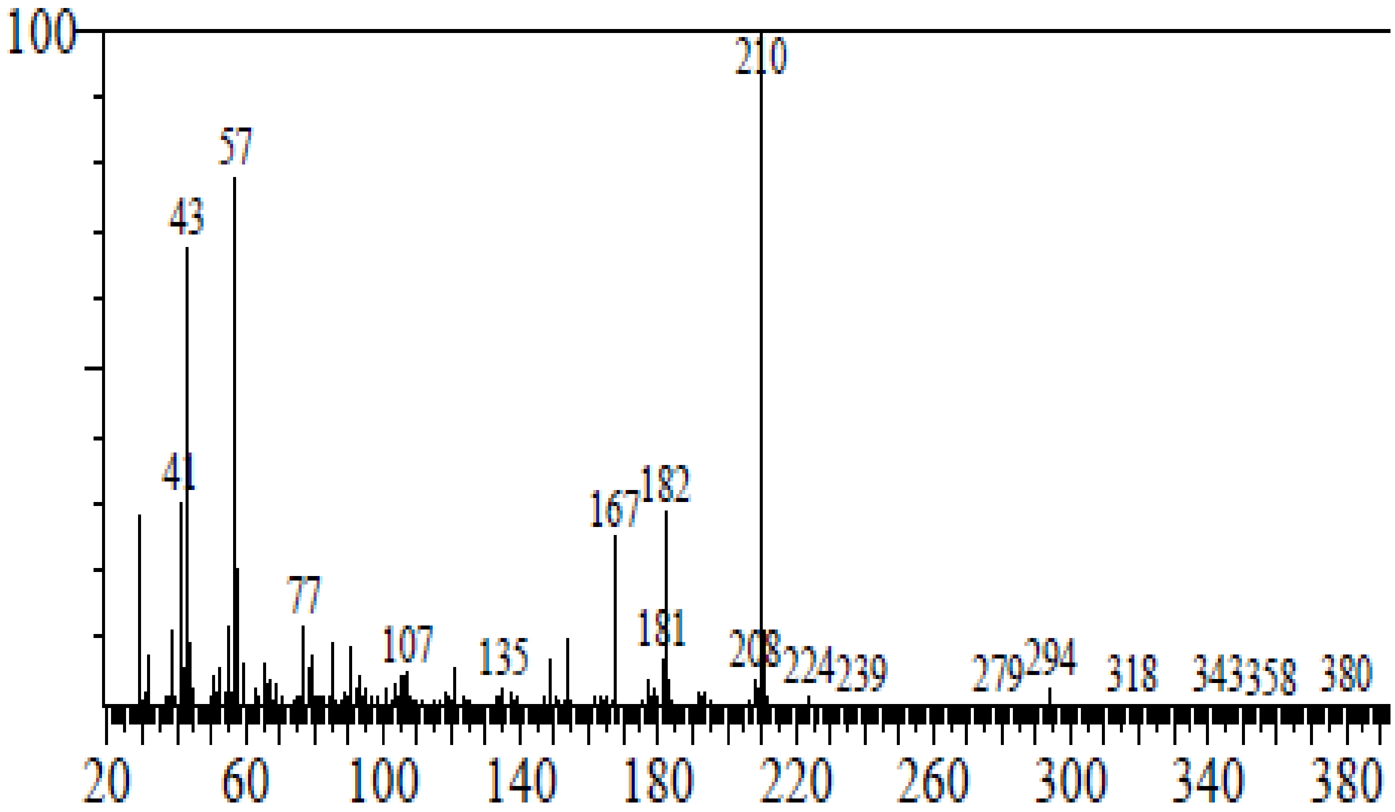

Figure S3. HRESIMS Spectrum of Dichrocephol **A** (**1**).

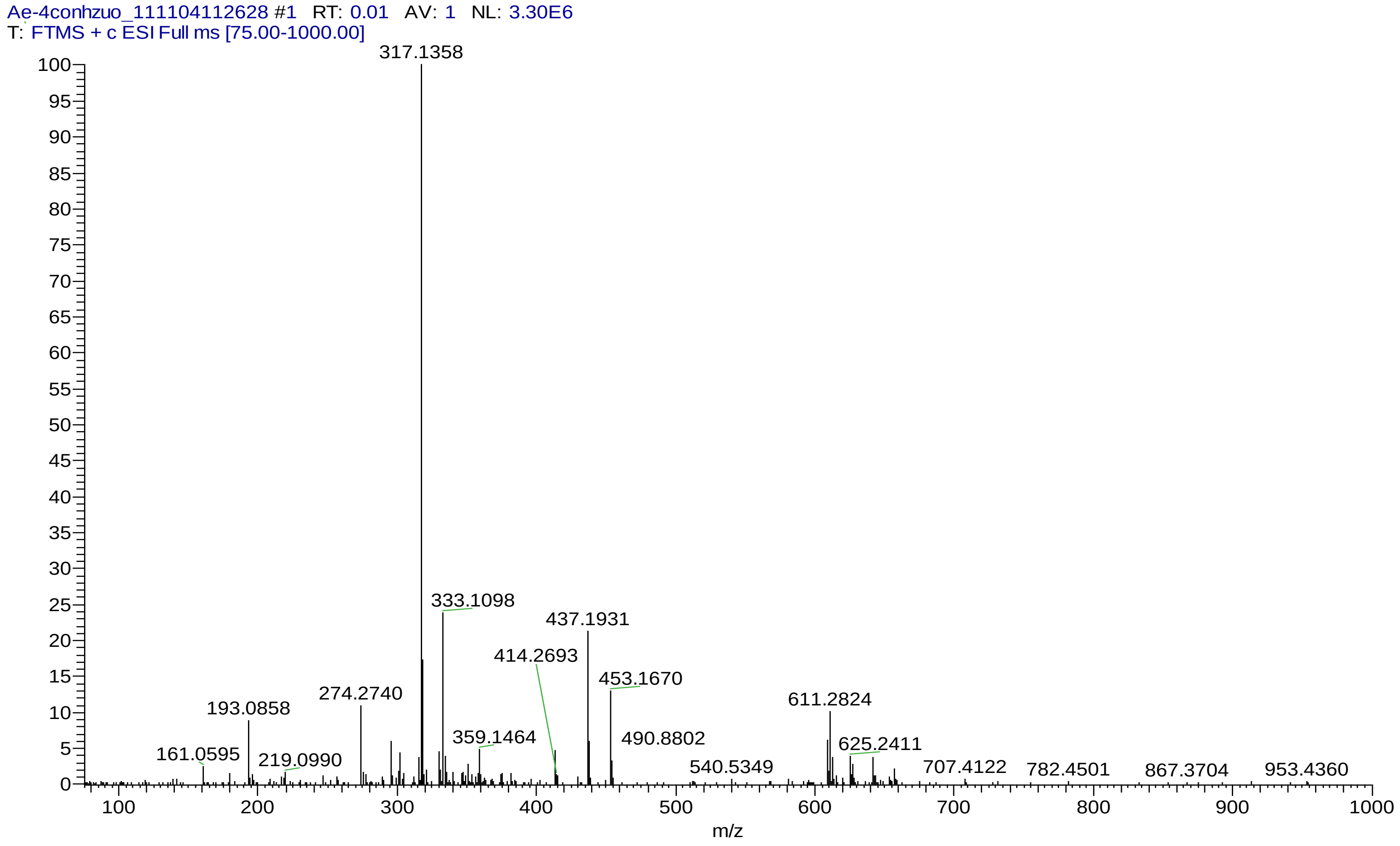

Figure S4. ^{1}H-NMR Spectrum of Dichrocephol **A** (**1**; 600 MHz, CD_3OD).

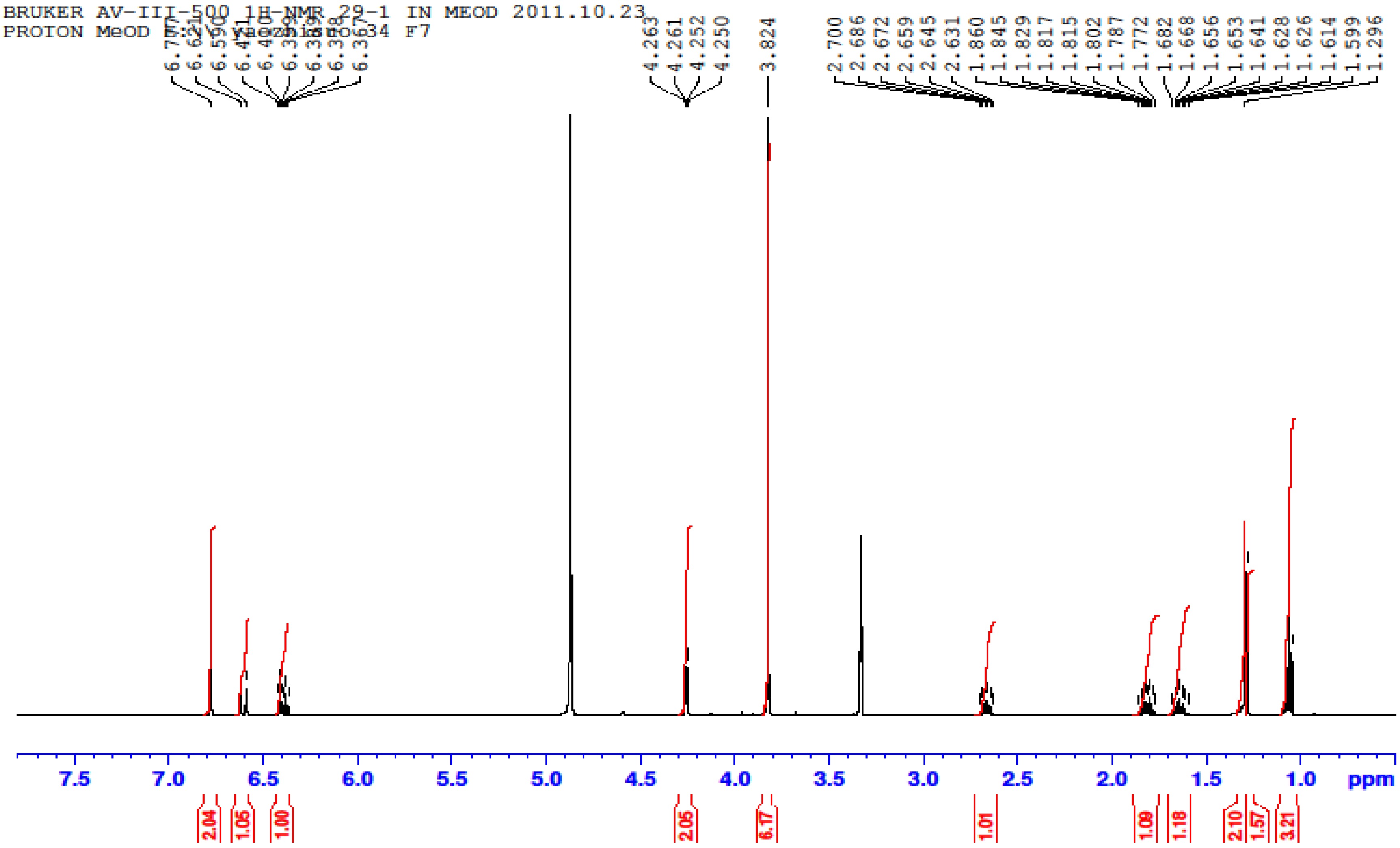

Figure S5. The magnified ^{1}H-NMR Spectrum of Dichrocephol **A** (**1**; 600 MHz, CD_3OD).

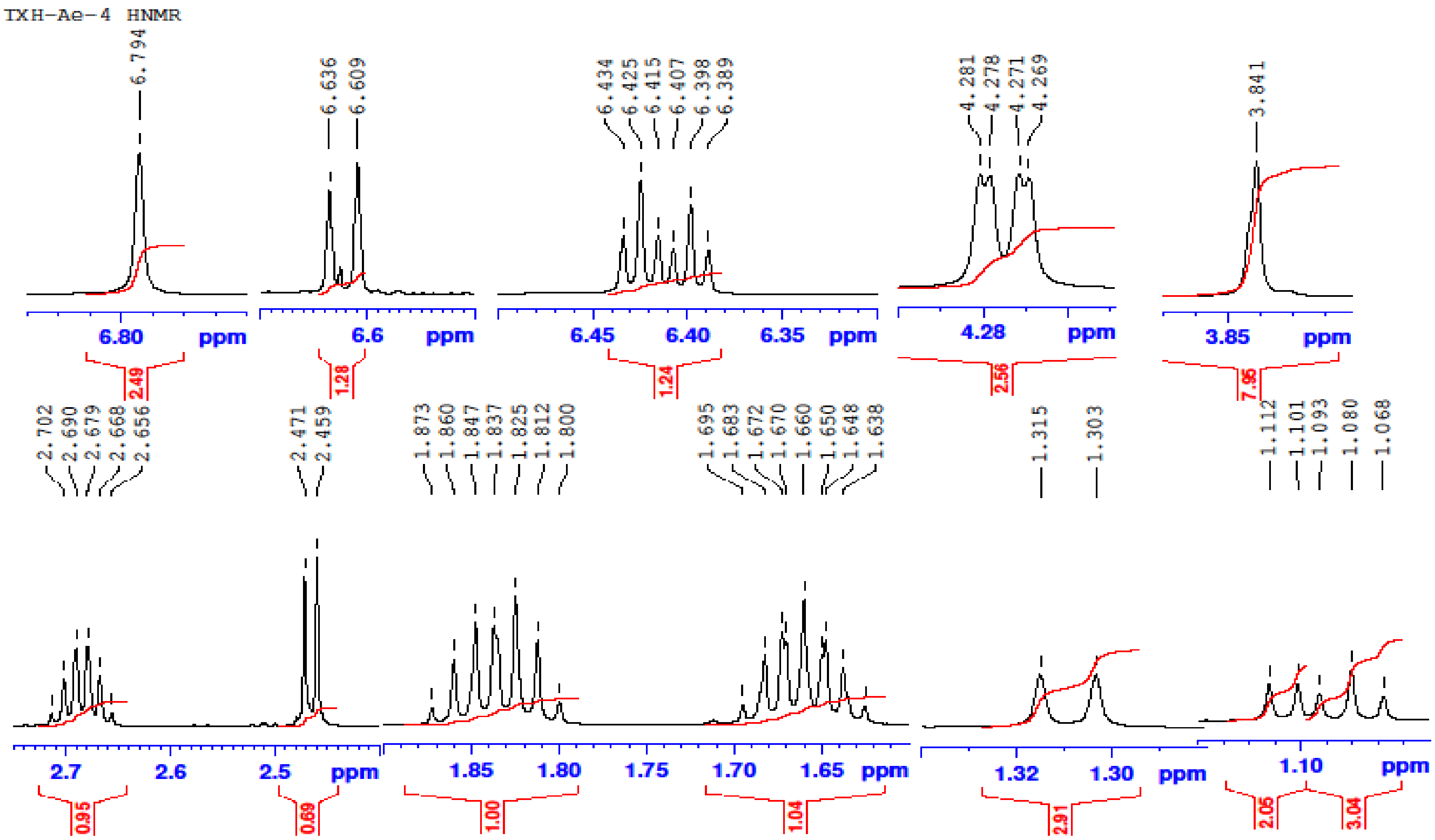

Figure S6. ^{13}C-NMR Spectrum of Dichrocephol **A (1**; 150 MHz, CD_3OD).

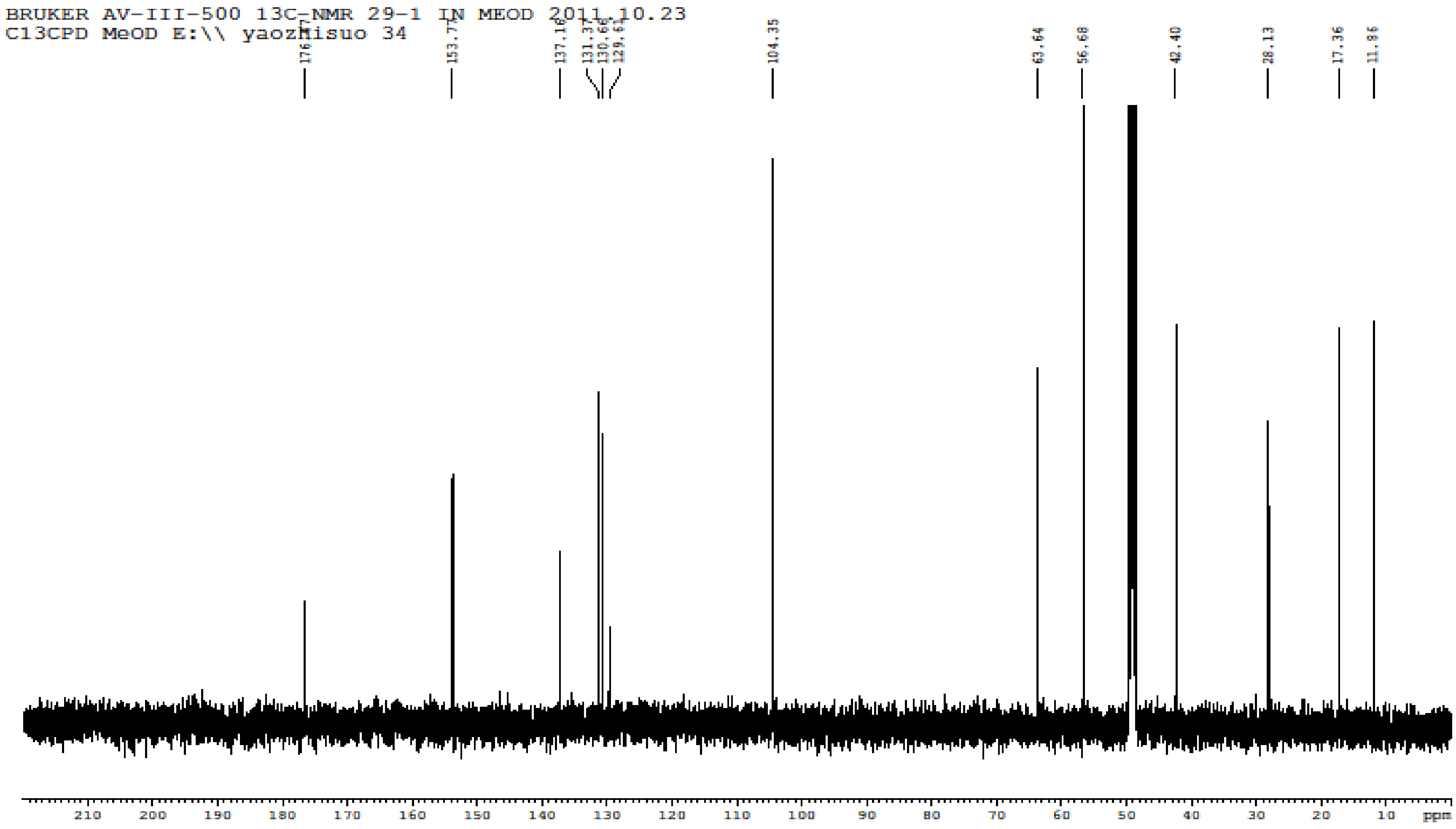

Figure S7. COSY Spectrum of Dichrocephol **A** (**1**; 600 MHz, CD_3OD).

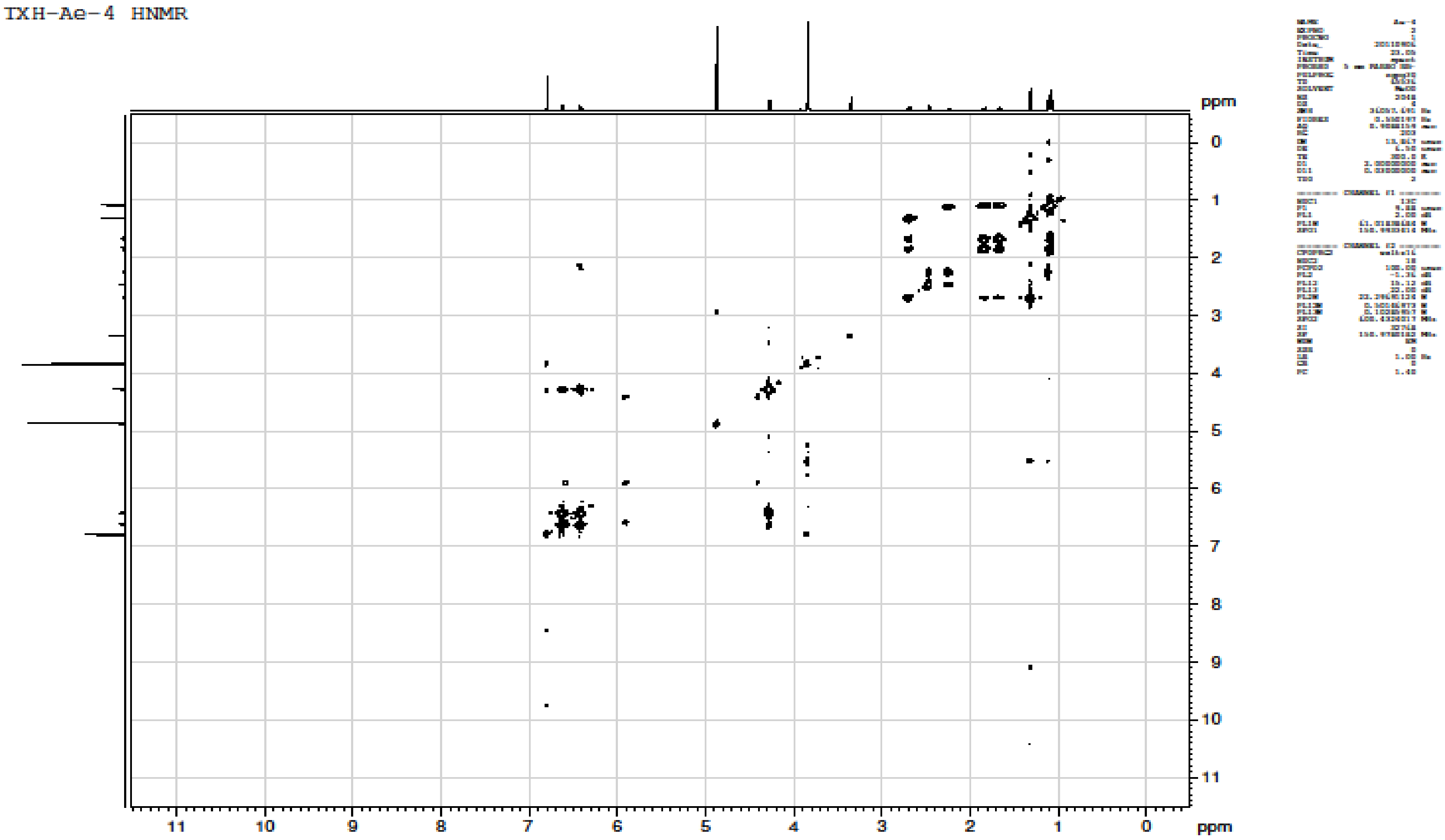

Figure S8. HSQC Spectrum of Dichrocephol **A (1**; 600 MHz, CD_3OD).

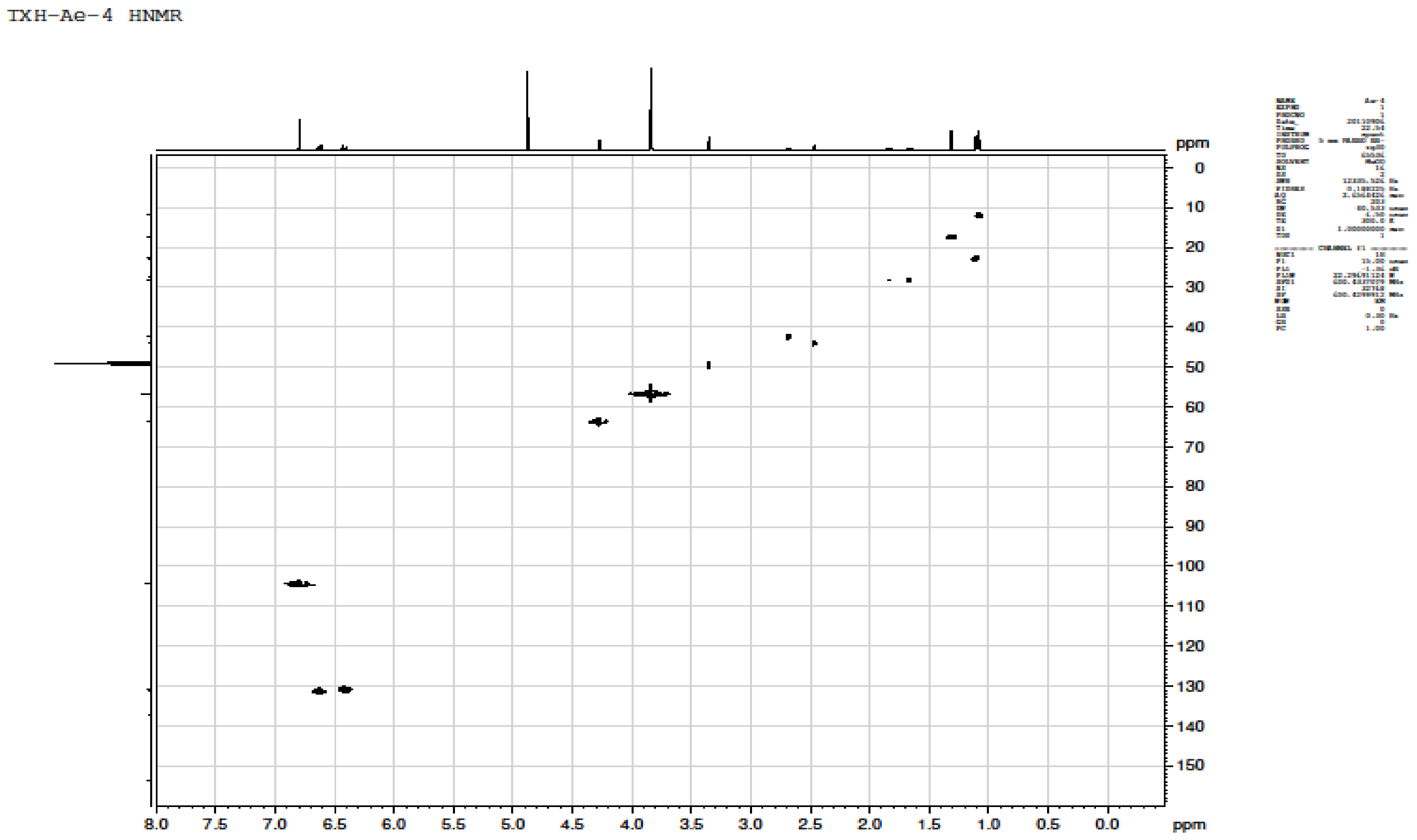

Figure S9. HMBC Spectrum of Dichrocephol **A** (**1**; 600 MHz, CD_3OD).

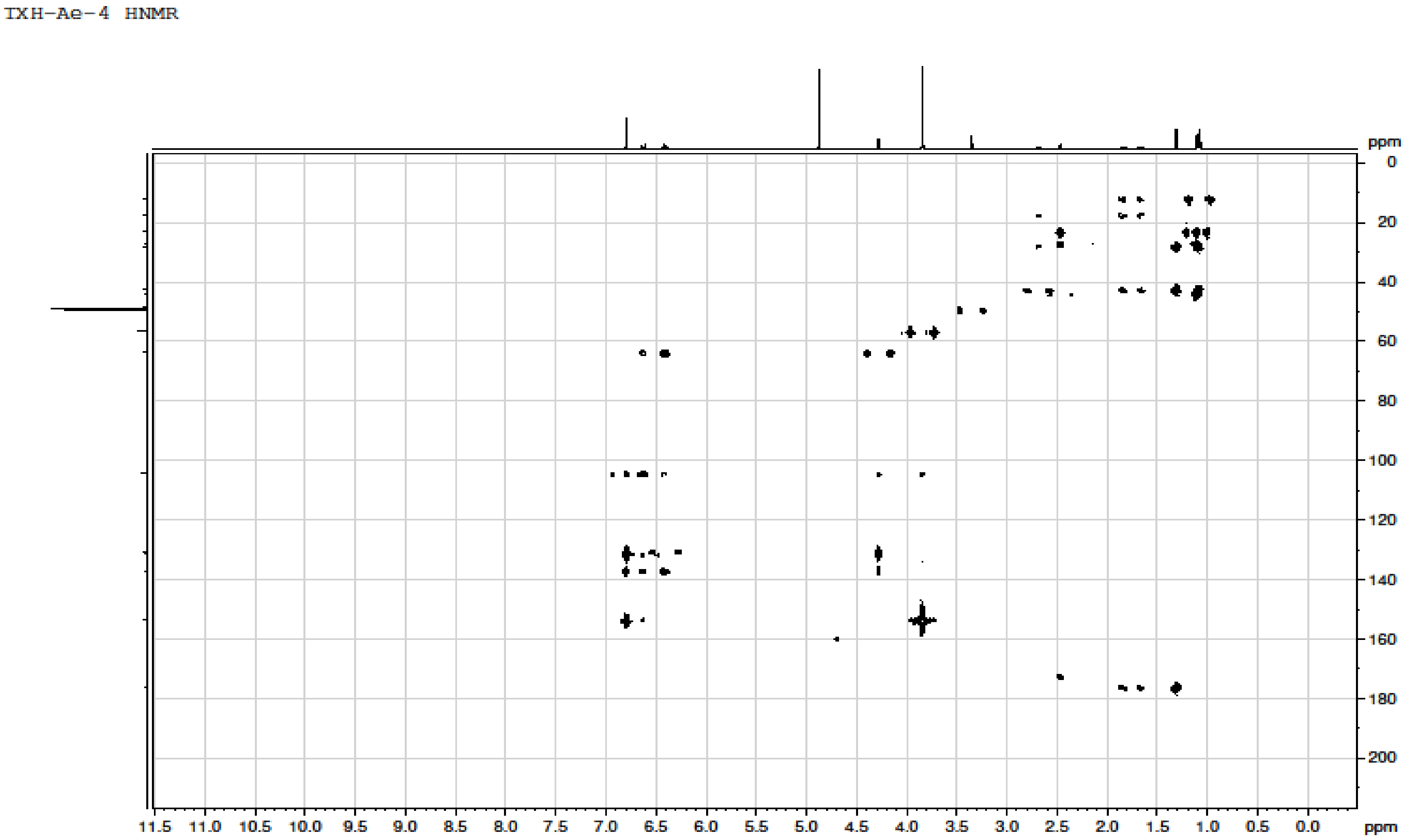

Figure S10. IR Spectrum of Dichrocephol **B** (**2**).

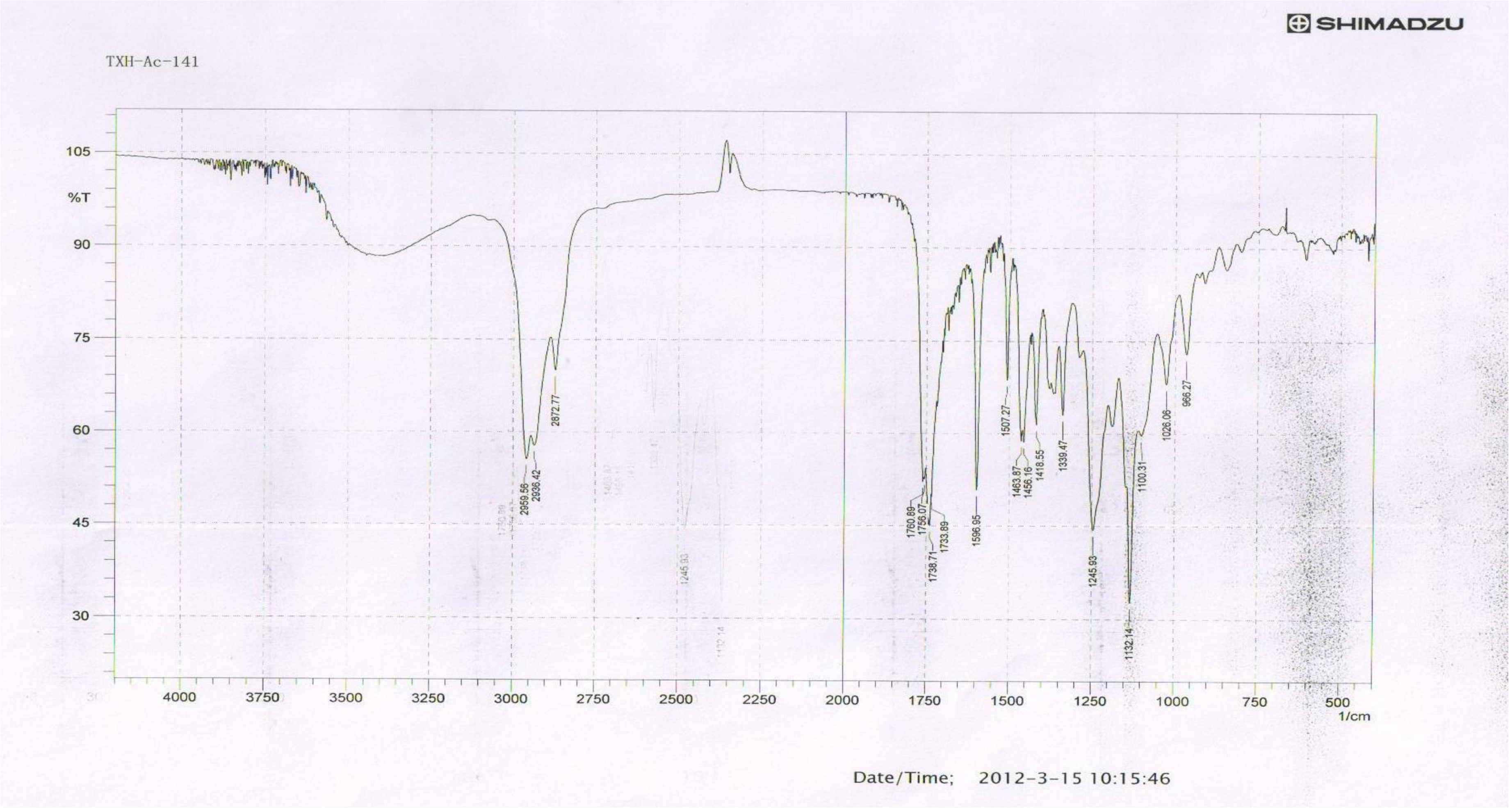

Figure S11. EIMS Spectrum of Dichrocephol **C** (**2**).

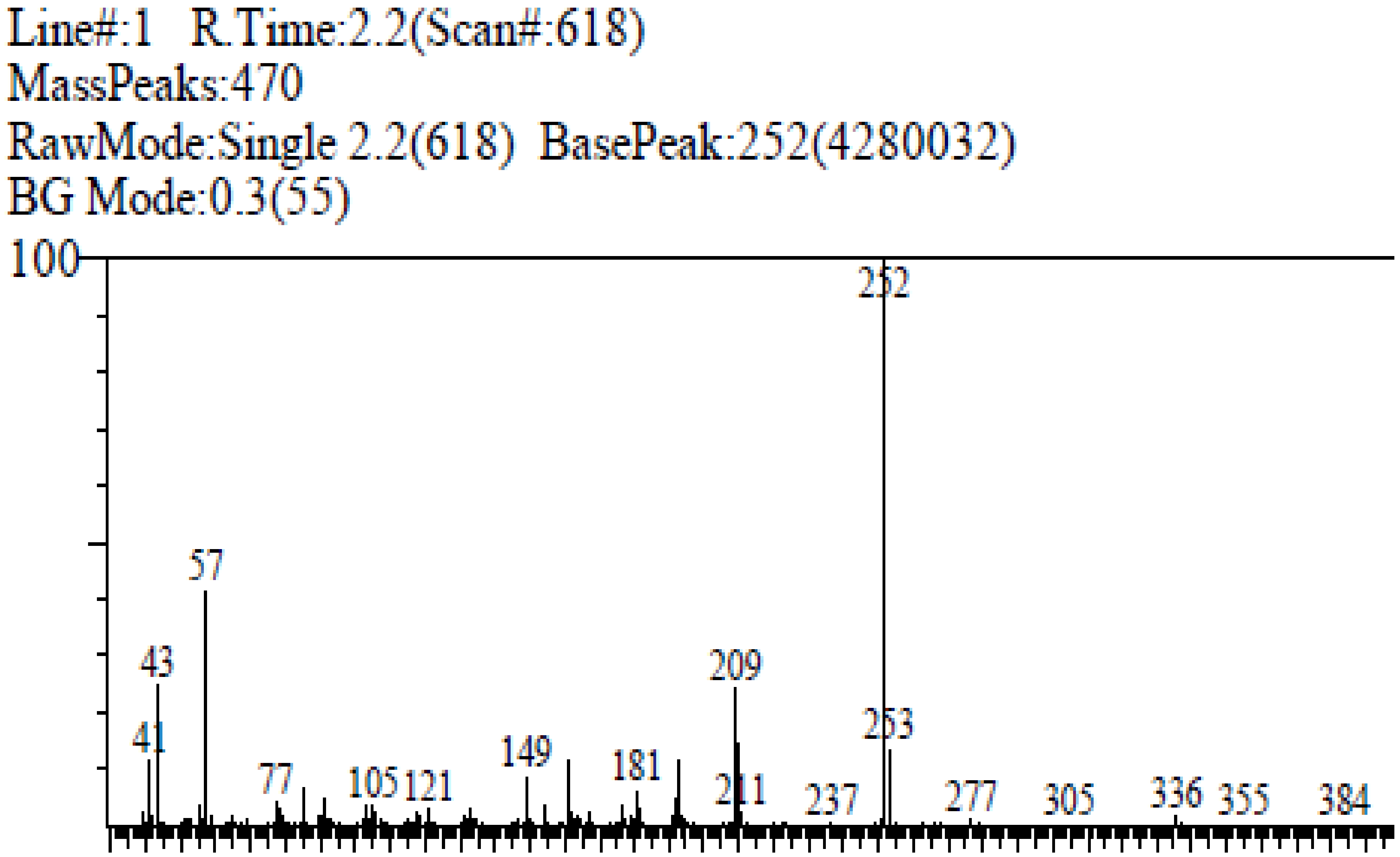

Figure S12. HRESIMS Spectrum of Dichrocephol **C** (**2**).

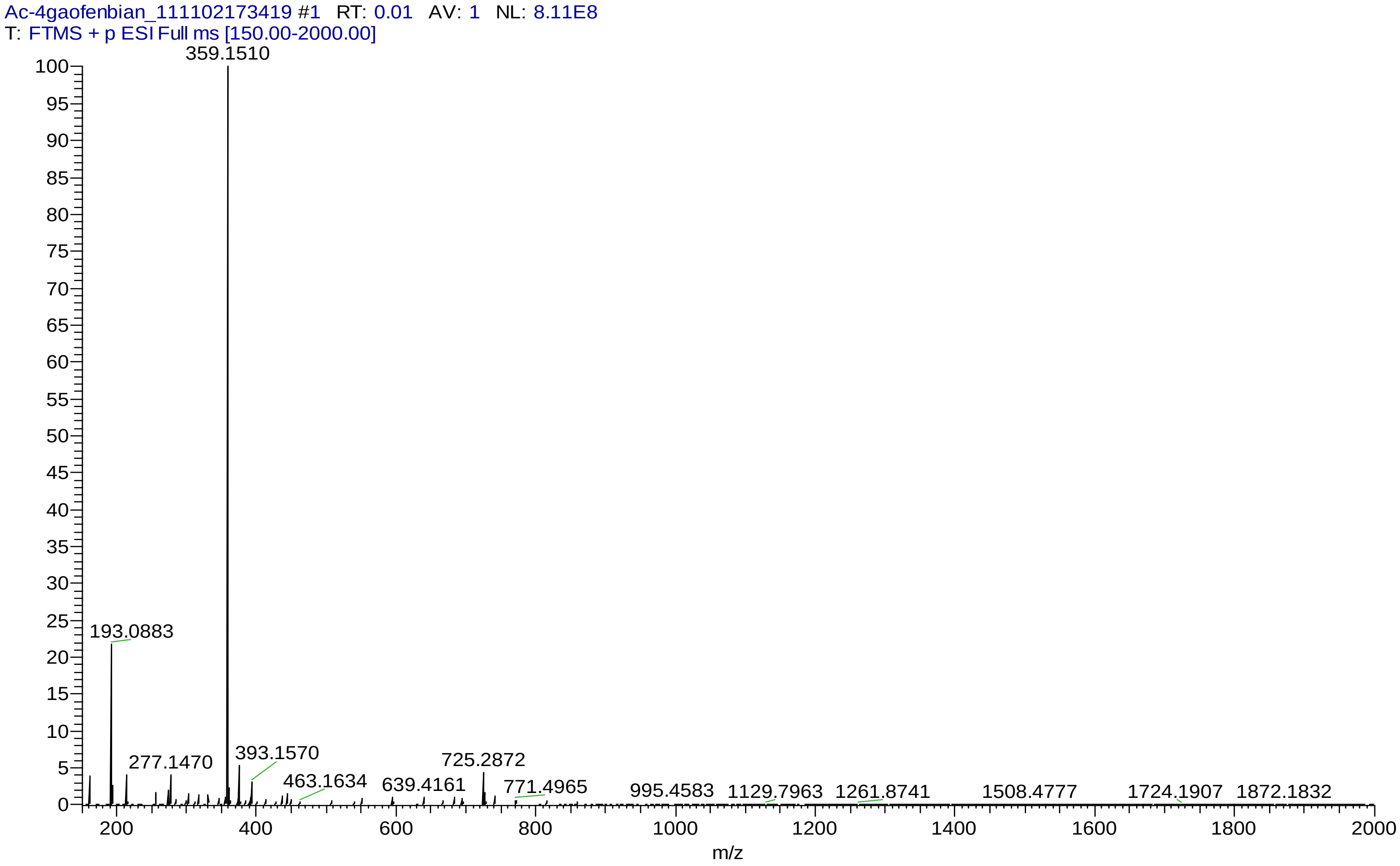

Figure S13. ^{1}H-NMR Spectrum of Dichrocephol **B** (**2**; 600 MHz, CD_3COCD_3).

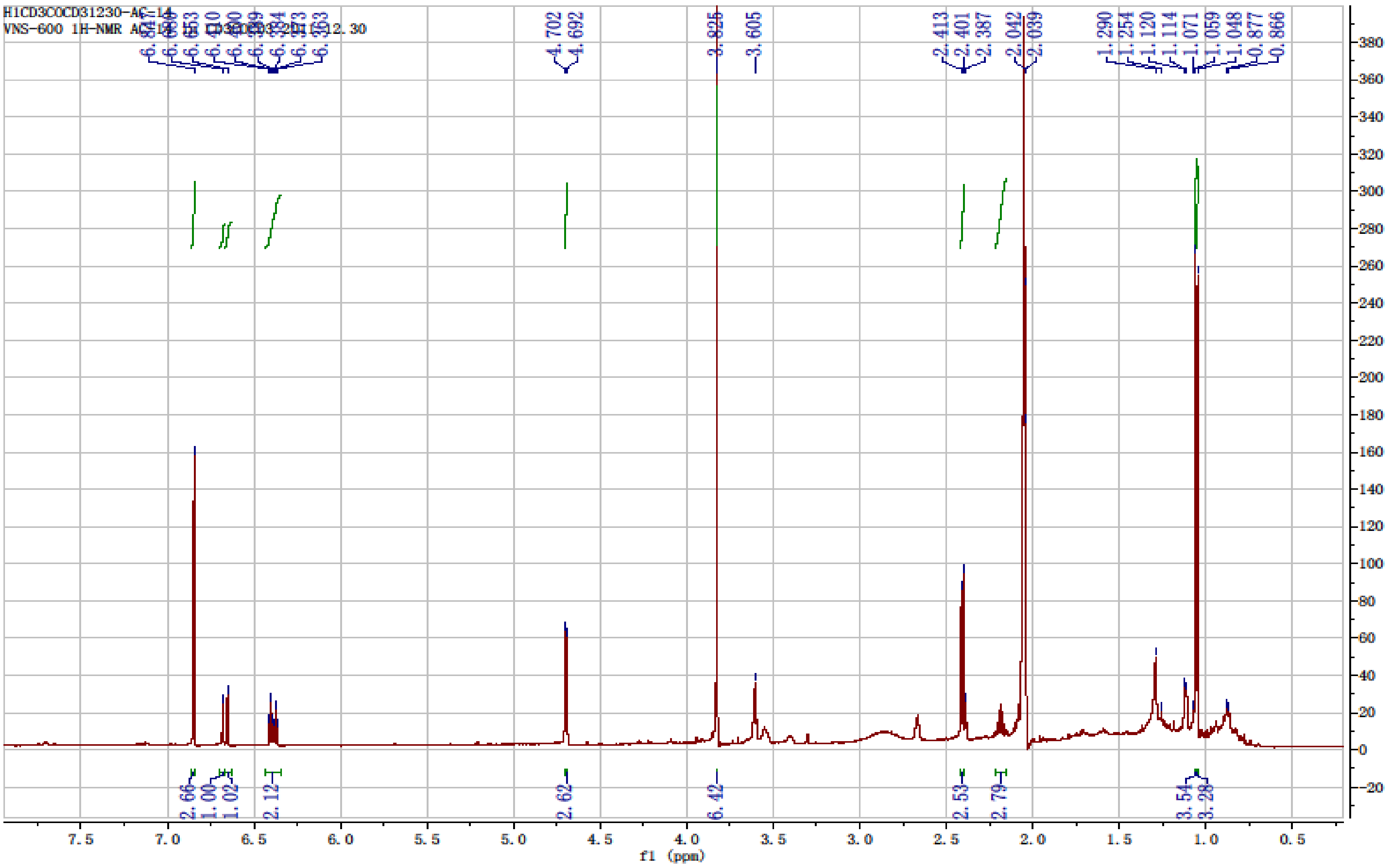

Figure S14. ^{13}C-NMR Spectrum of Dichrocephol **B** (**2**; 150 MHz, CD_3COCD_3).

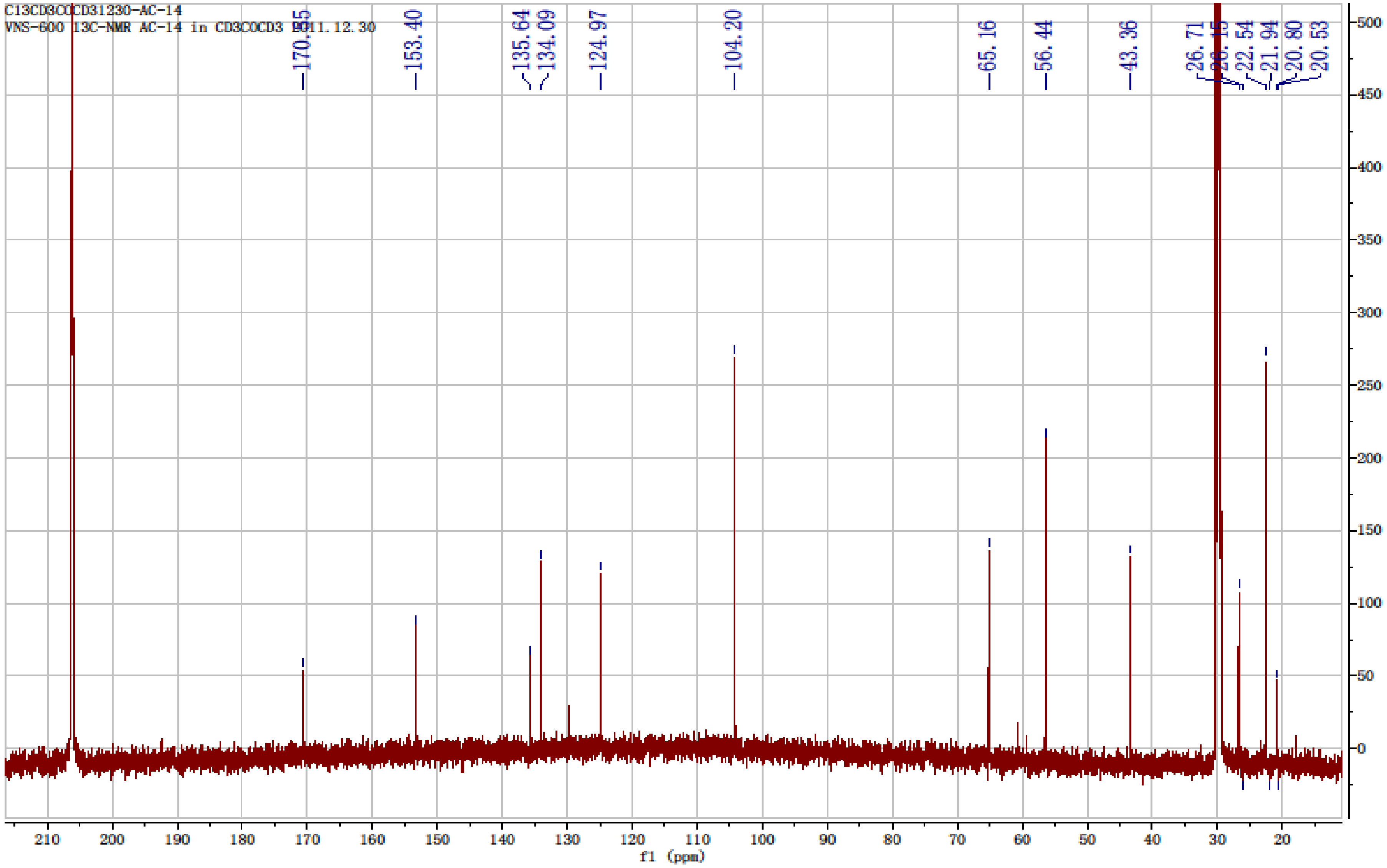

Figure S15. IR Spectrum of Dichrocephol **C** (**3**).

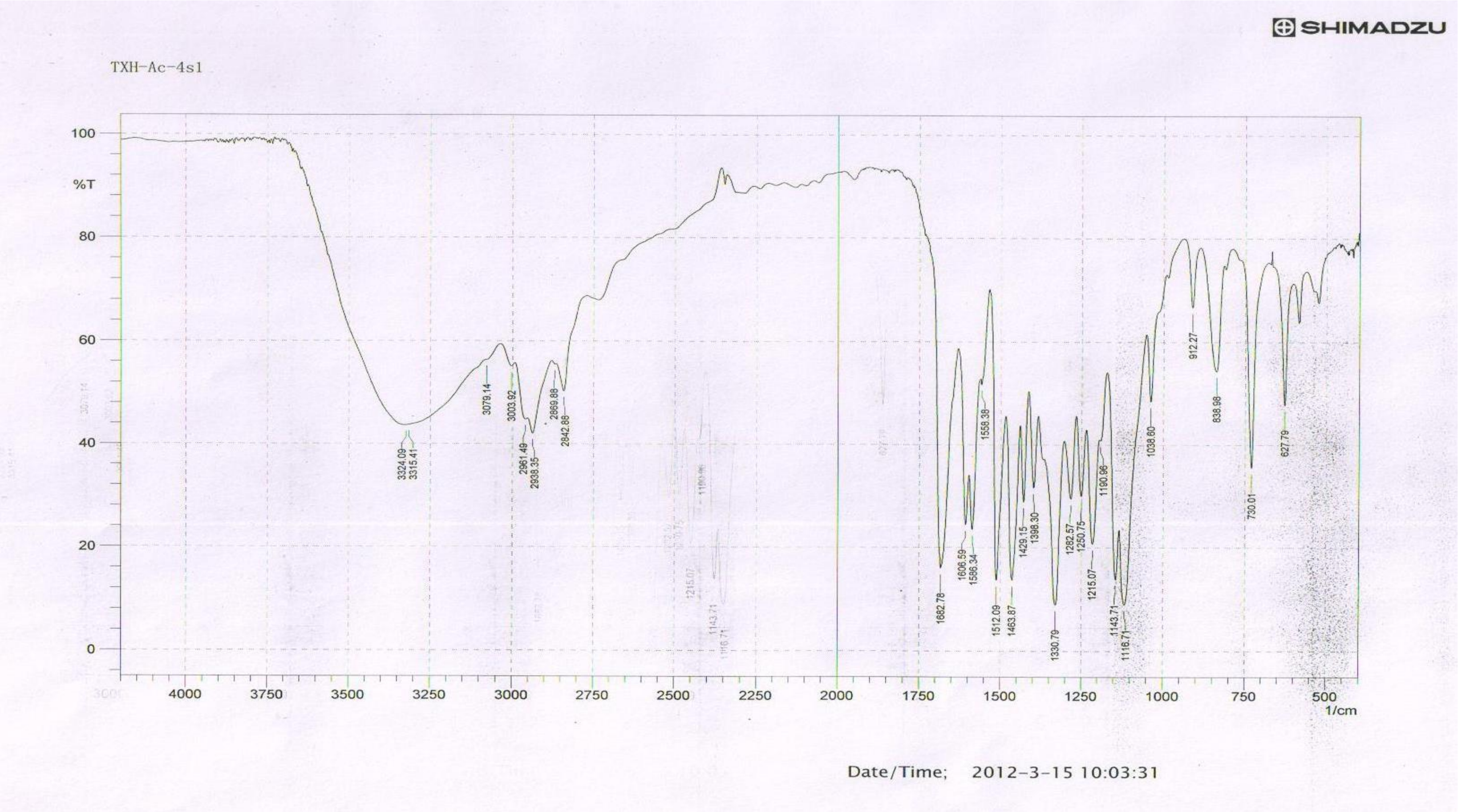

Figure S16. EIMS Spectrum of Dichrocephol **C** (**3**).

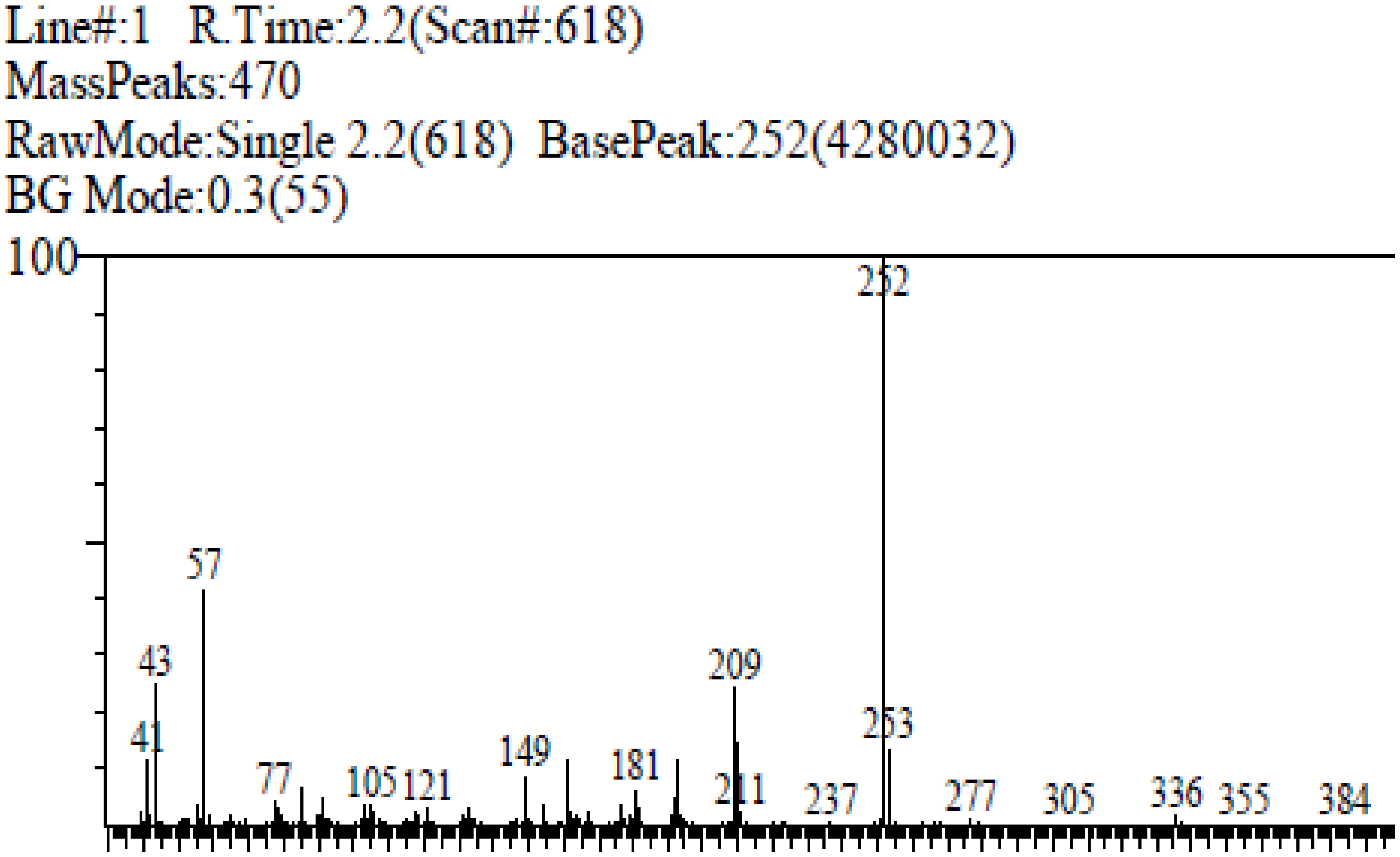

Figure S17. HRESIMS Spectrum of Dichrocephol **C** (**3**).

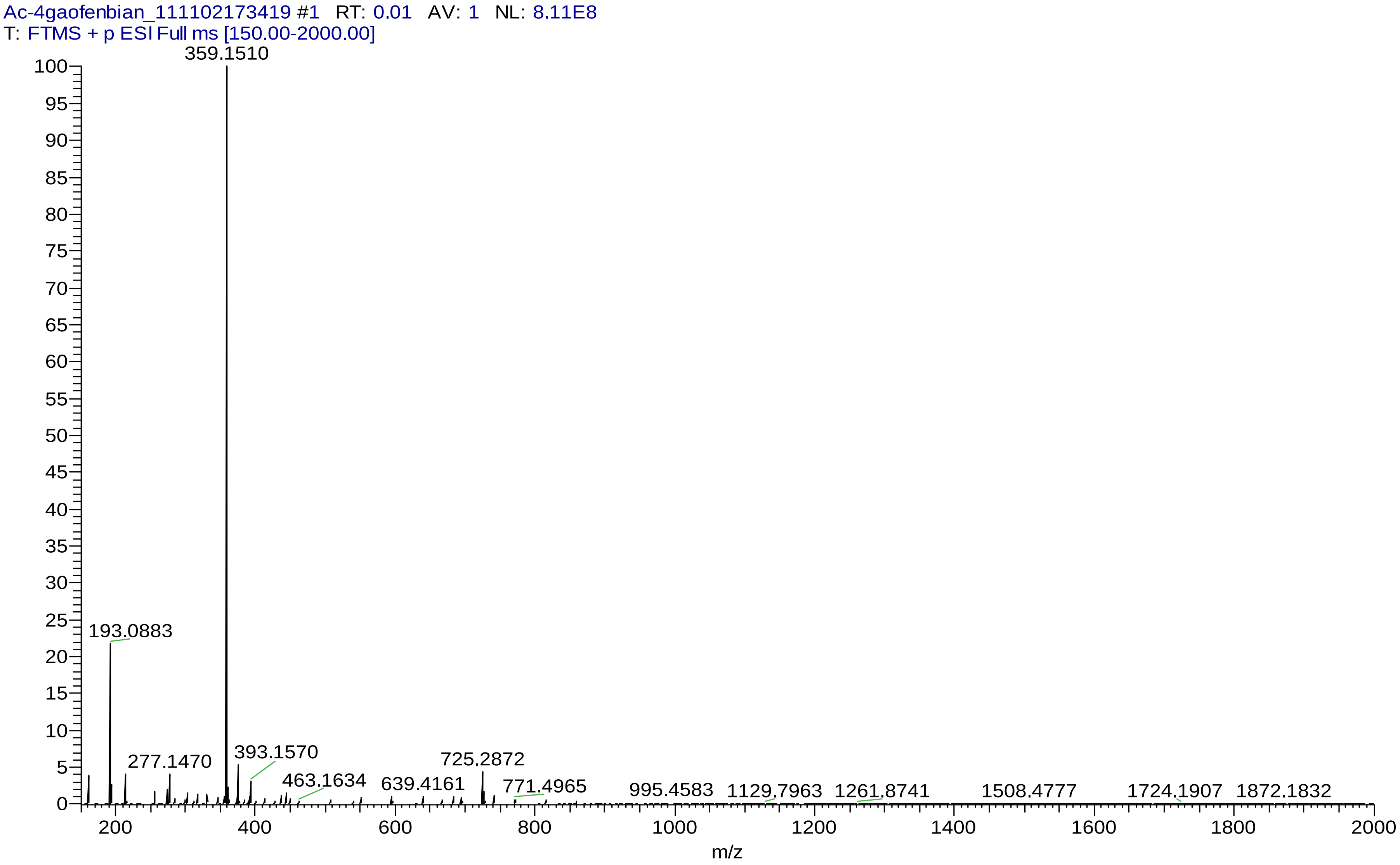

Figure S18. ^{1}H-NMR Spectrum of Dichrocephol **B** and **C** (**2**, **3**; 600 MHz, CD_3COCD_3).

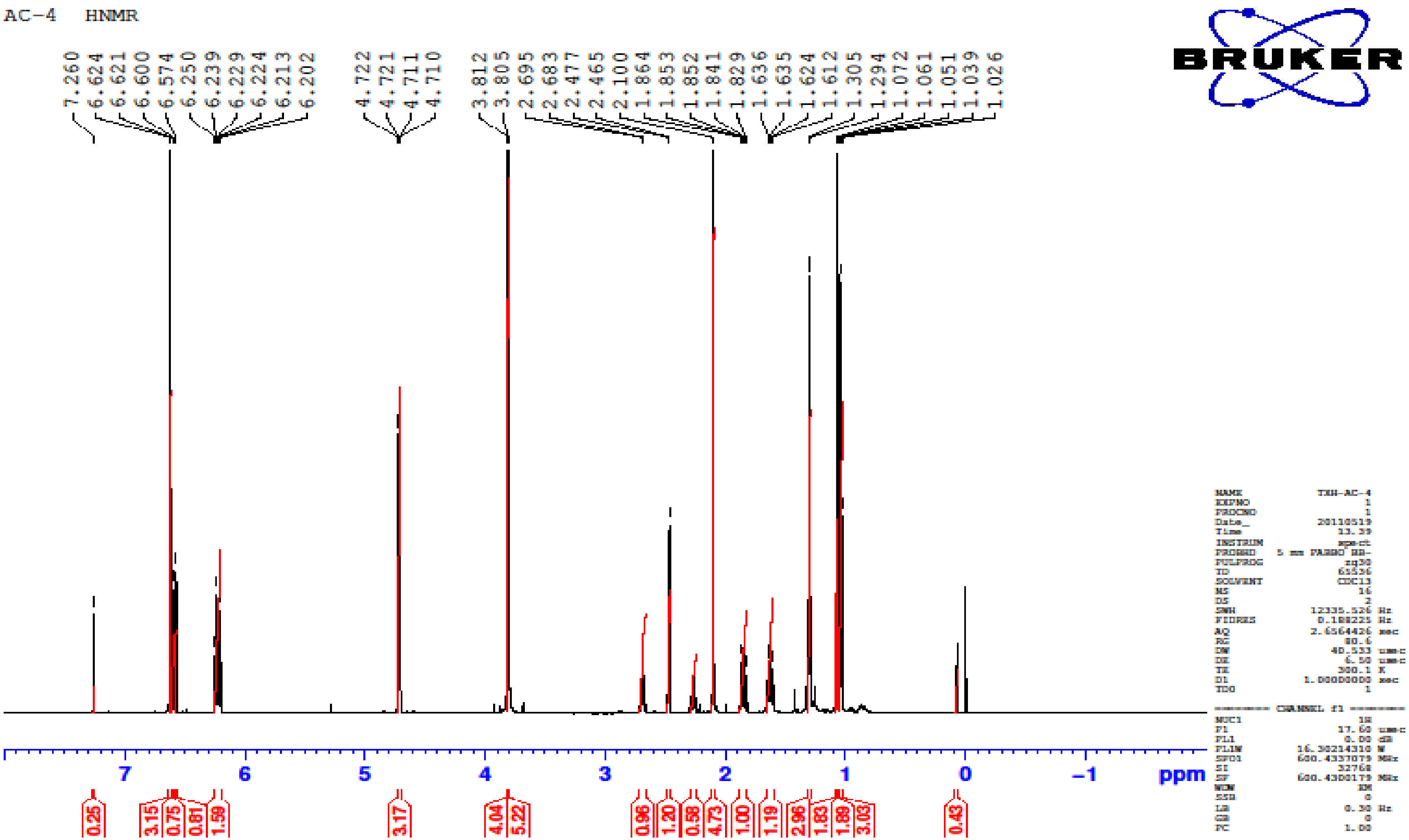

Figure S19. ^{13}C-NMR Spectrum of Dichrocephol **B** and **C** (**2**, **3**; 150 MHz, CD_3COCD_3).

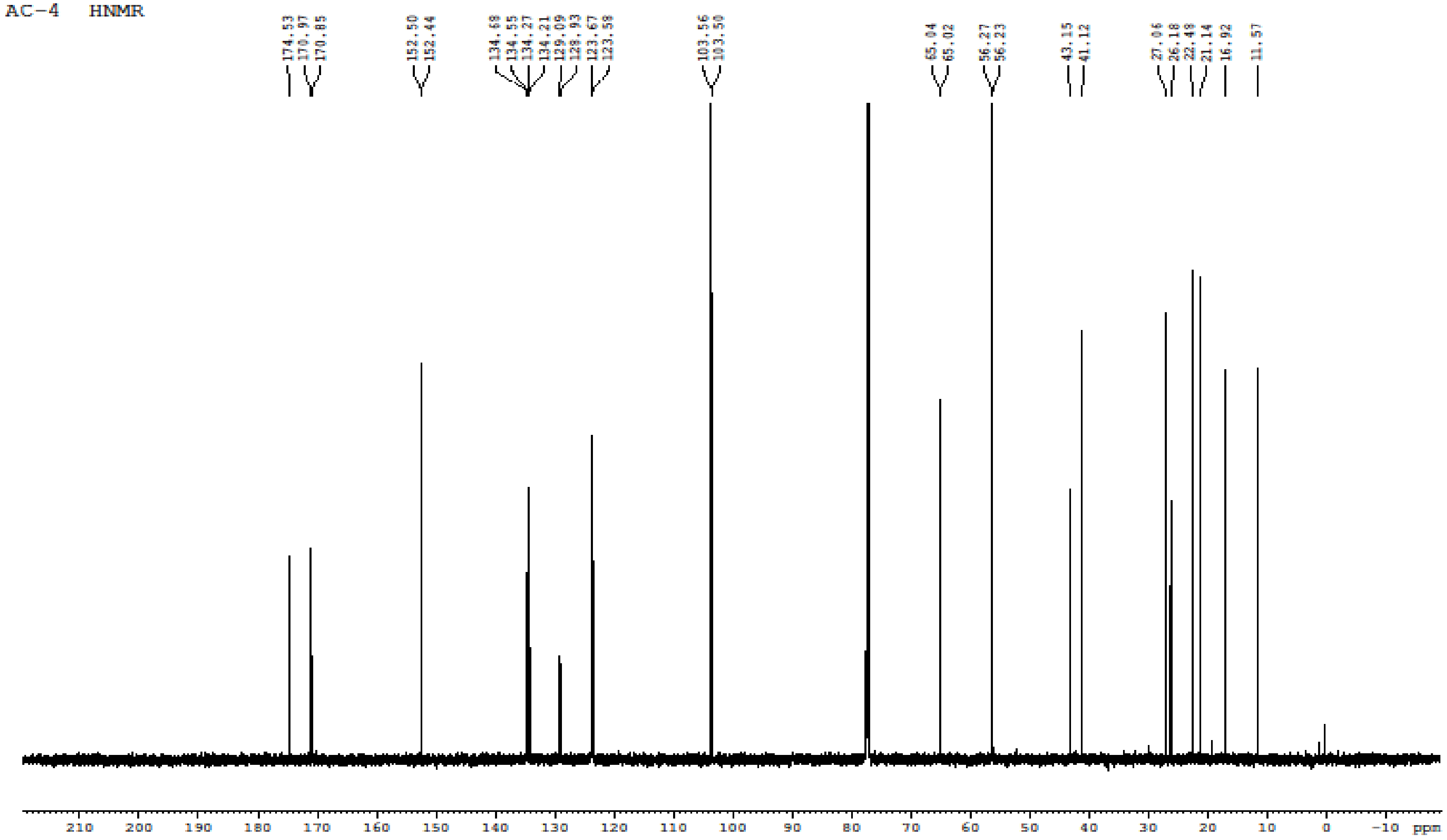

Figure S20. DEPT Spectrum of Dichrocephol **B** and **C** (**2**, **3**; 150 MHz, CD_3COCD_3).

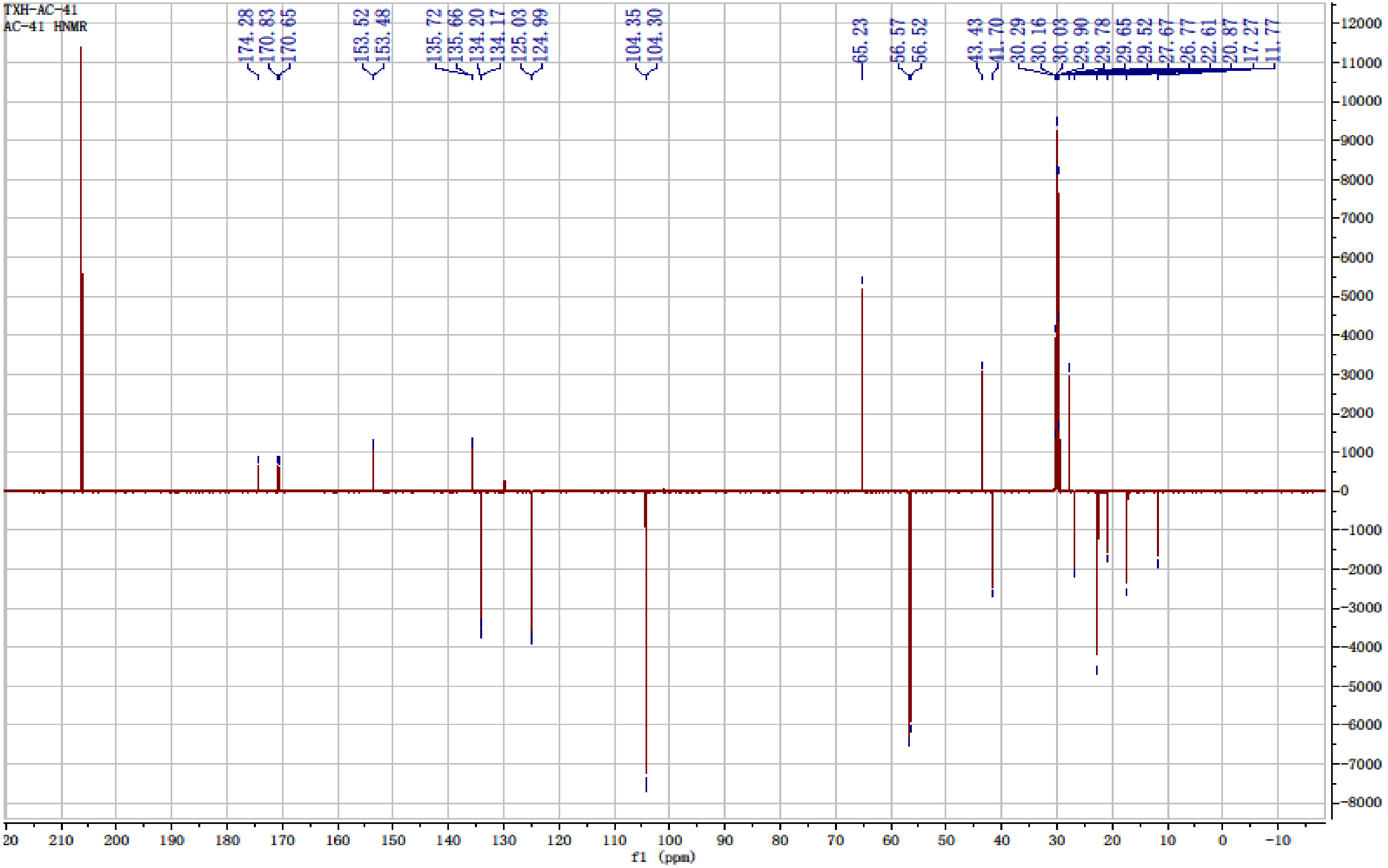

Figure S21. HSQC Spectrum of Dichrocephol **B** and **C** (**2**, **3**; 600 MHz, CD_3COCD_3).

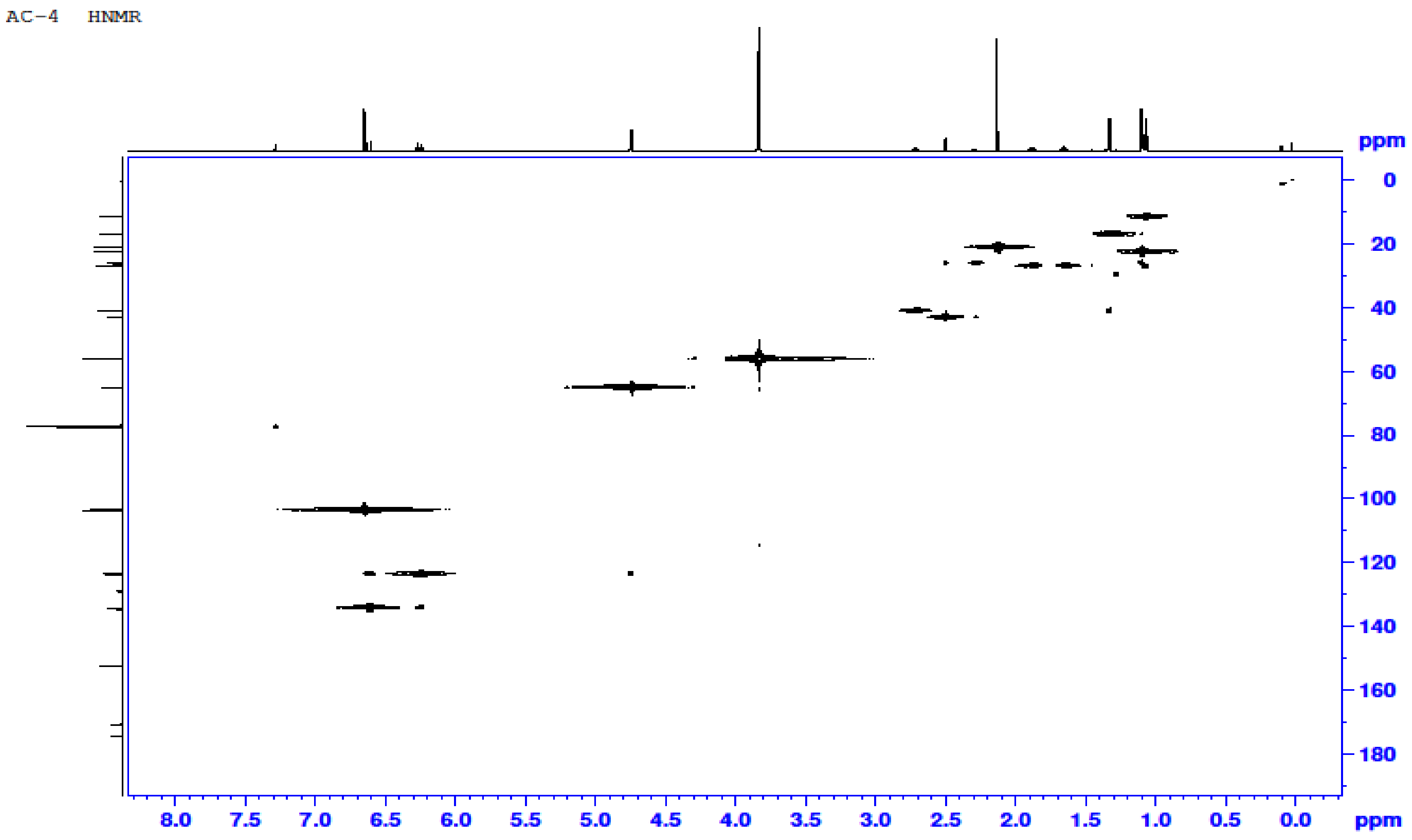

Figure S22. HMBC Spectrum of Dichrocephol **B** and **C** (**2**, **3**; 600 MHz, CD_3COCD_3).

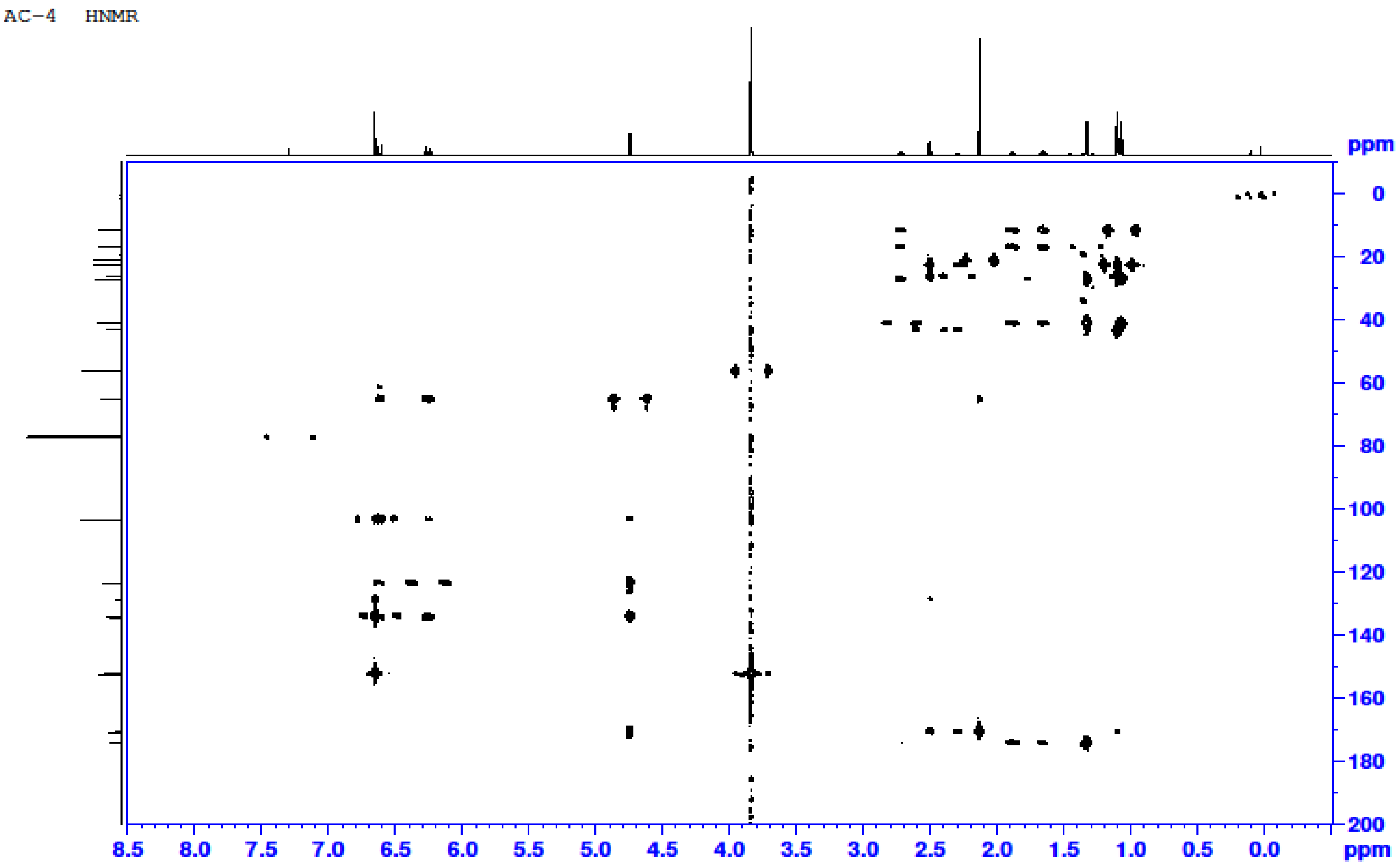

Figure S23. IR Spectrum of Dichrocephol **D** (**4**).

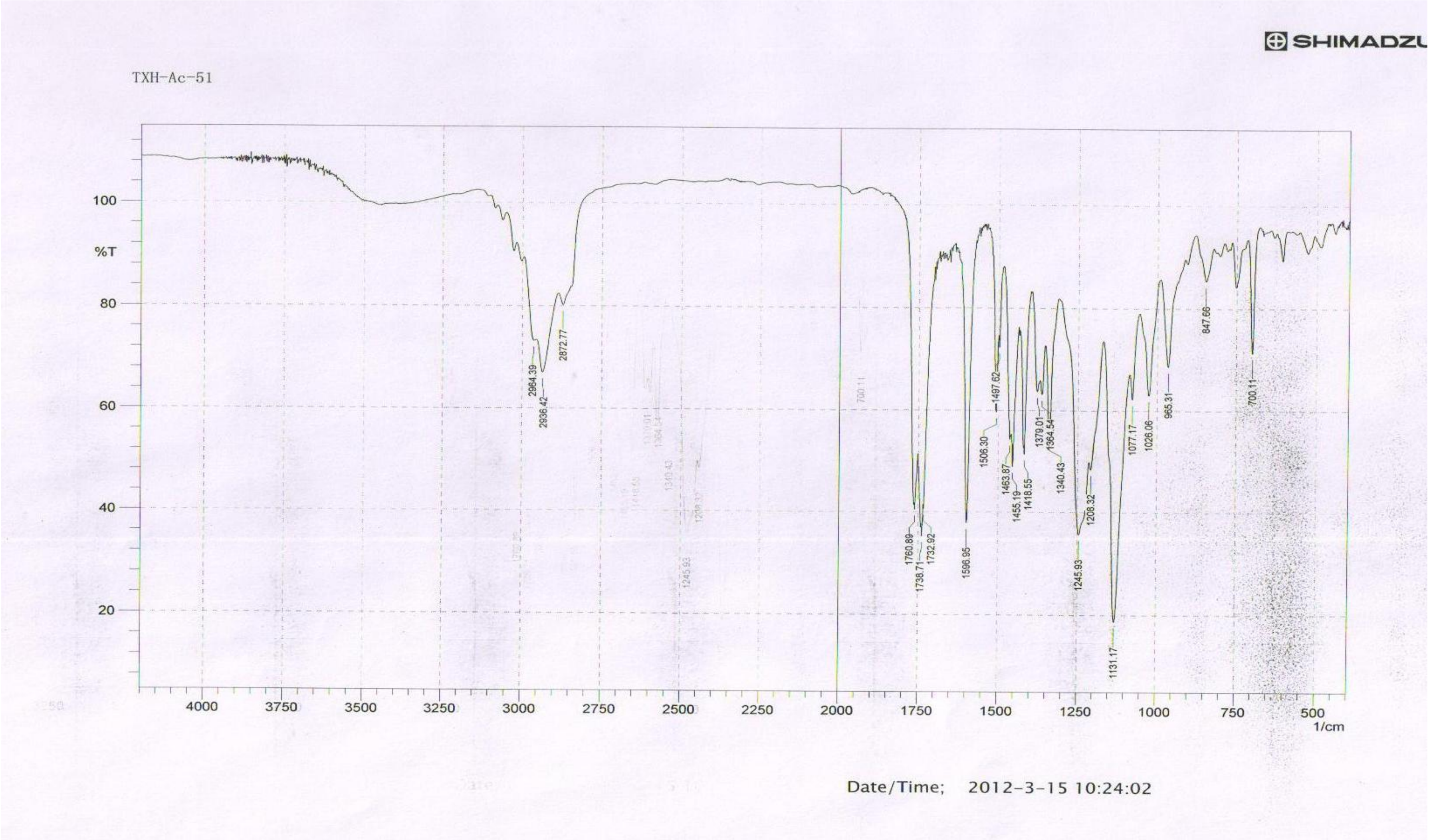

Figure S24. EIMS Spectrum of Dichrocephol **D** (**4**).

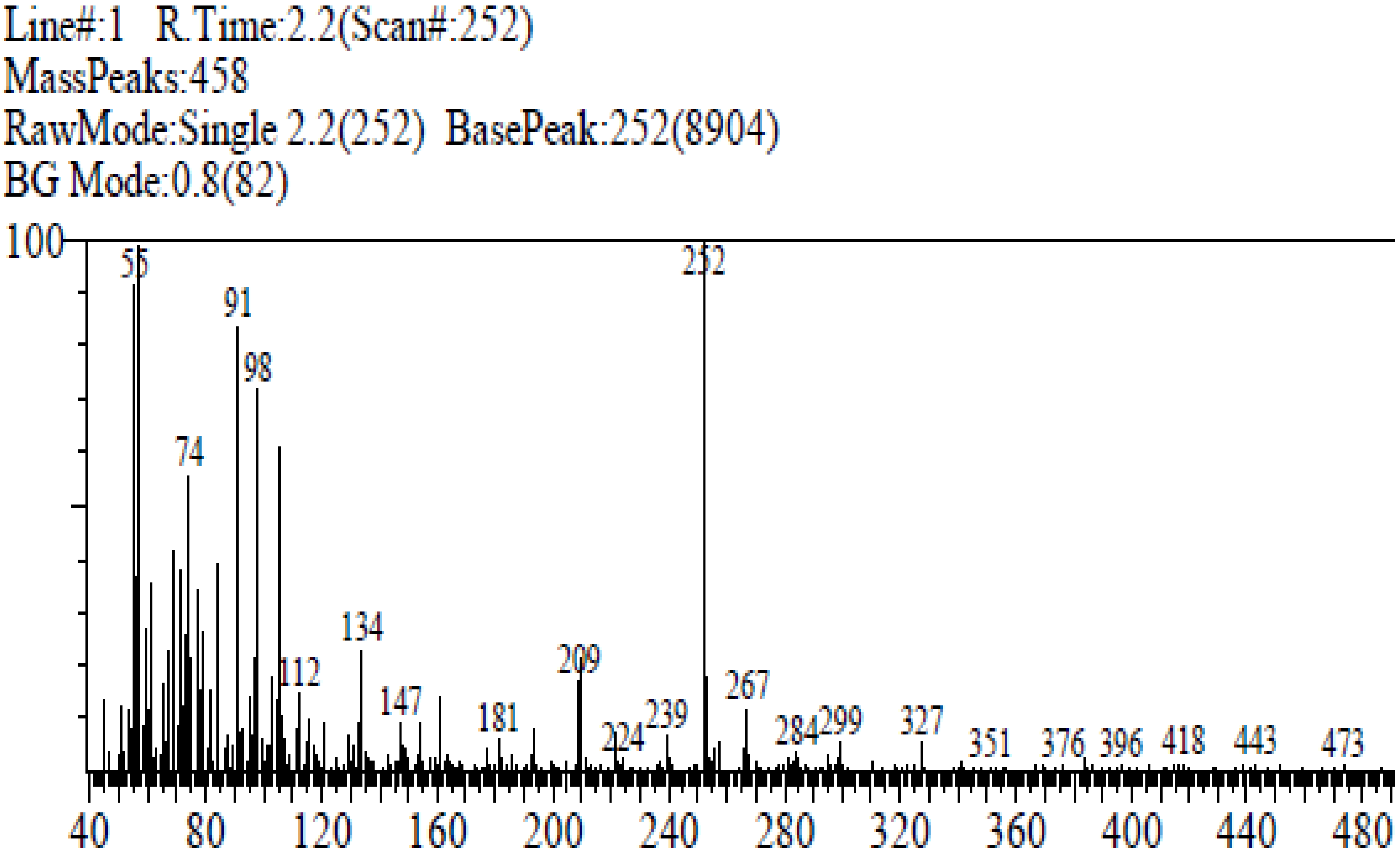

Figure S25. HRESIMS Spectrum of Dichrocephol **D** (**4**).

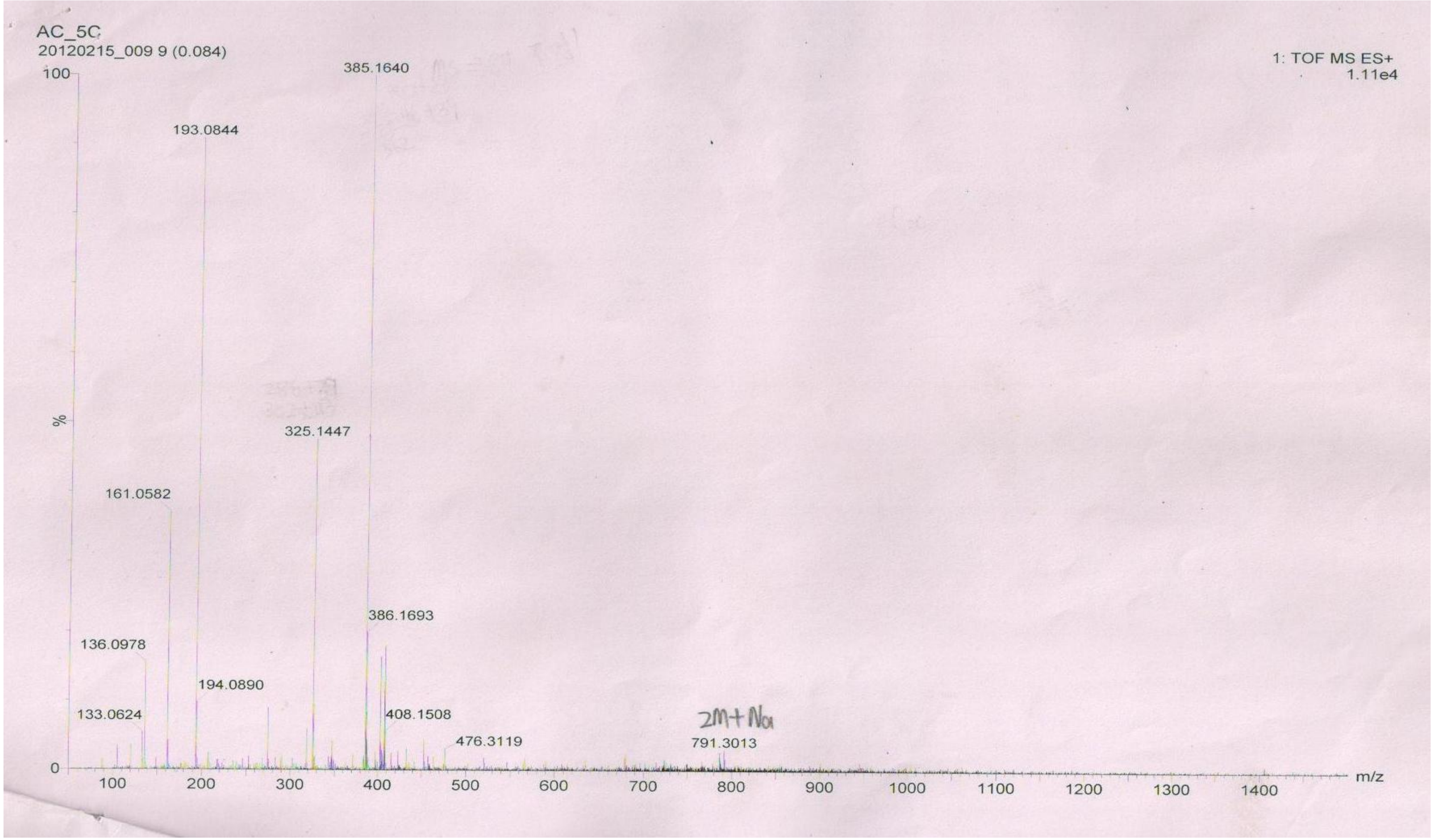

Figure S26. ^{1}H-NMR Spectrum of Dichrocephol **D** (**4**; 500 MHz, CD_3COCD_3).

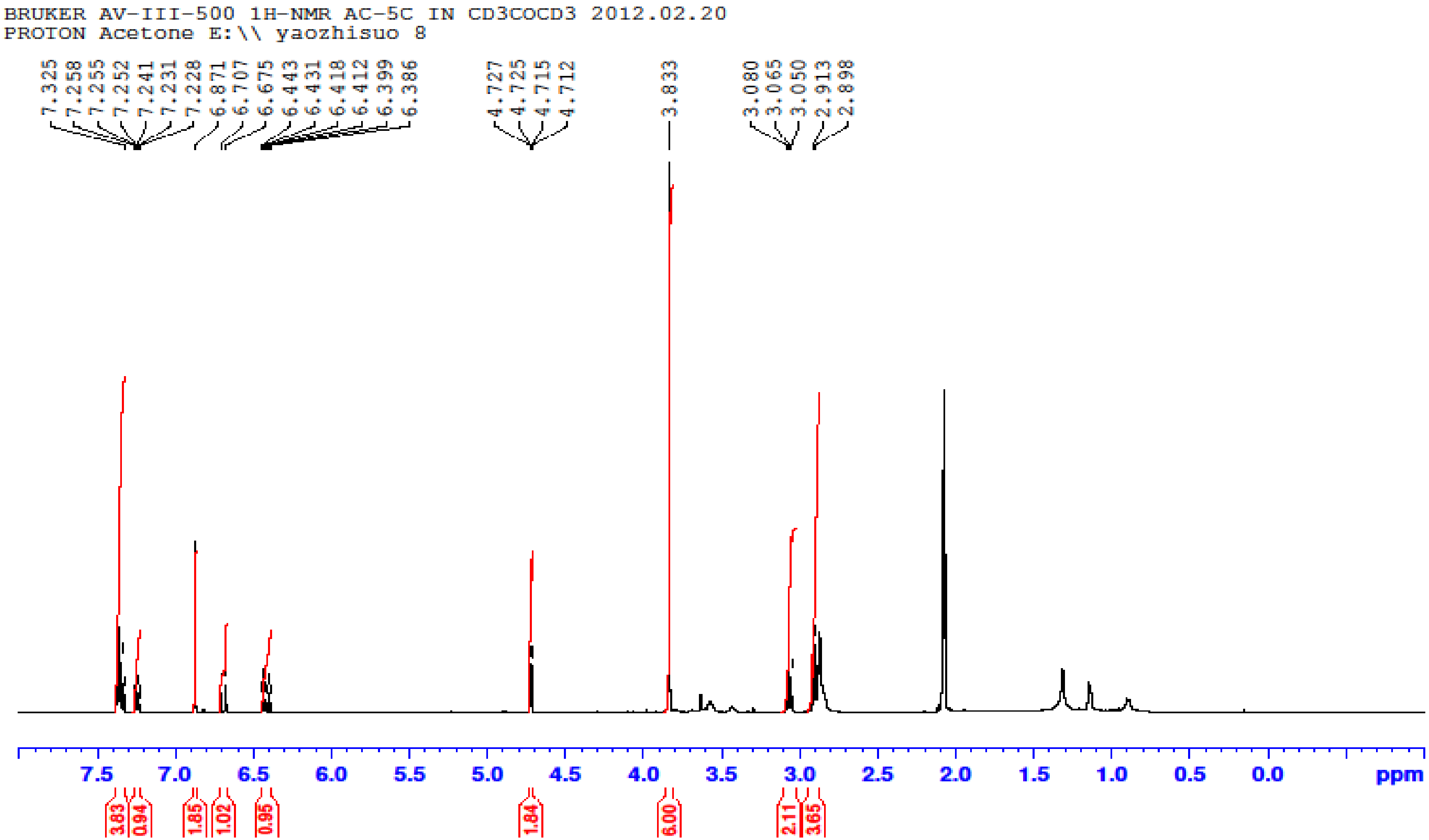

Figure S27. The magnified ^{1}H-NMR Spectrum of Dichrocephol **D** (**4**; 500 MHz, CD_3COCD_3).

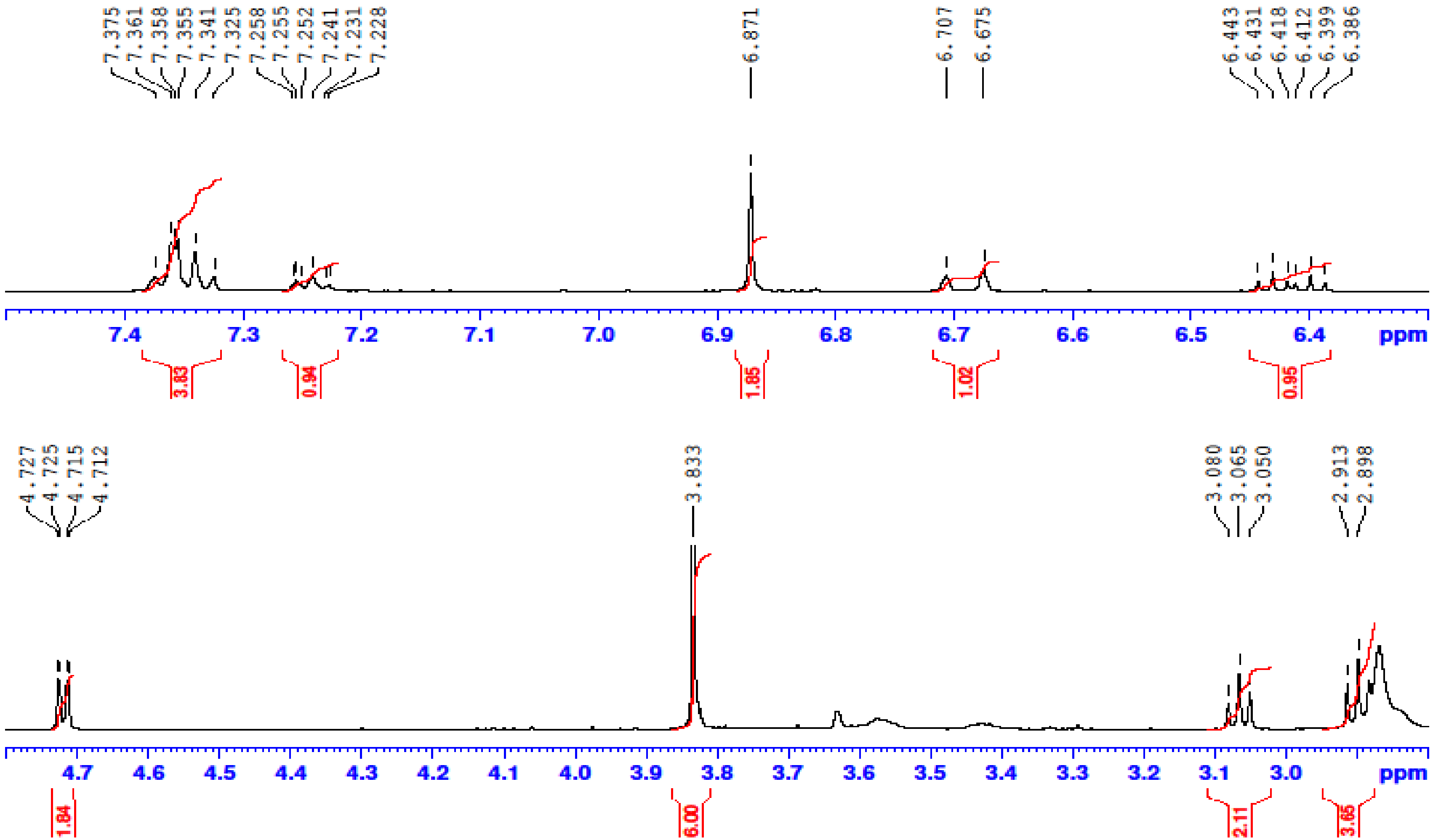

Figure S28. ^{13}C-NMR Spectrum of Dichrocephol **D** (**4**; 125 MHz, CD_3COCD_3).

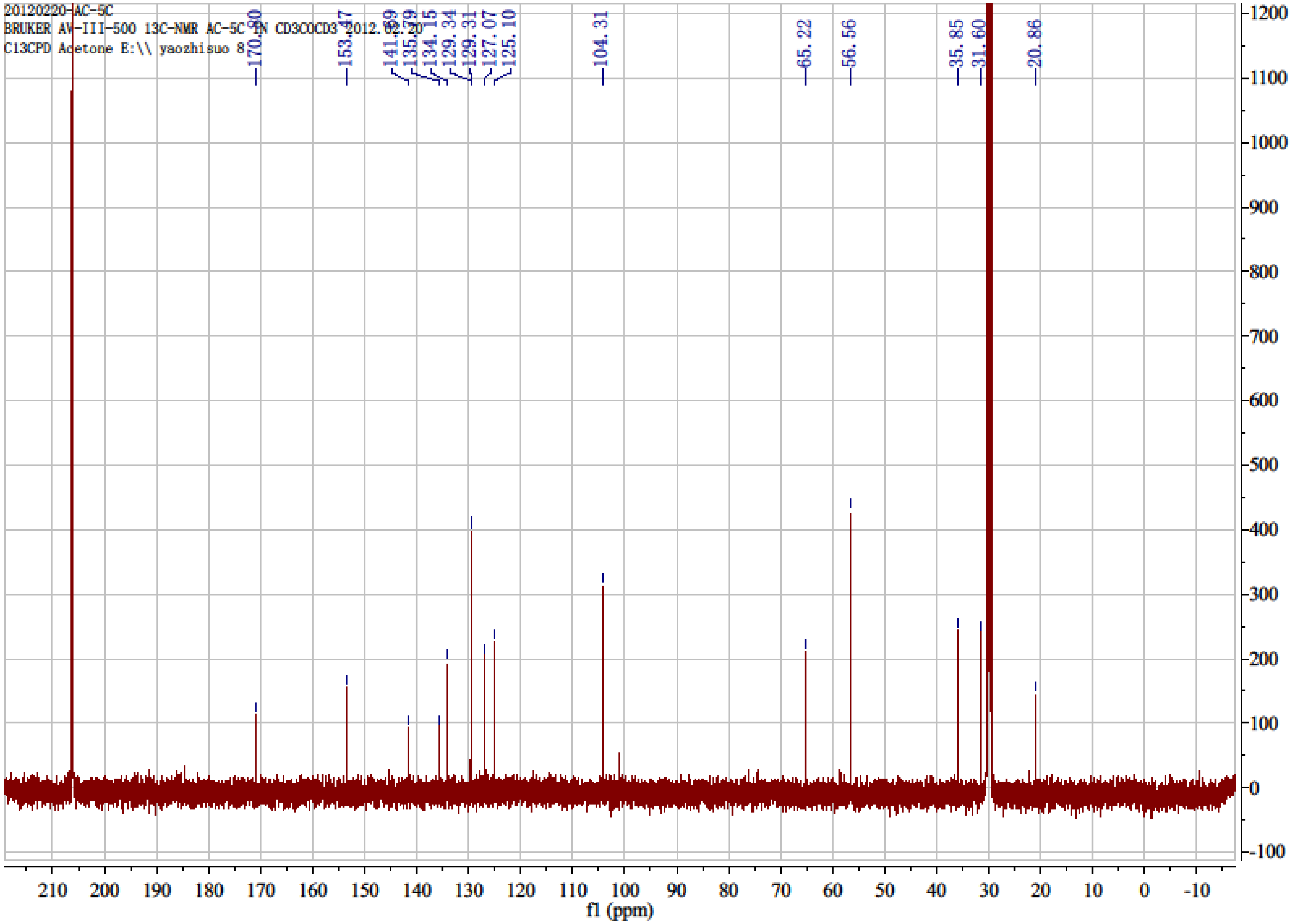

Figure S29. COSY Spectrum of Dichrocephol **D** (**4**; 500 MHz, CD_3COCD_3).

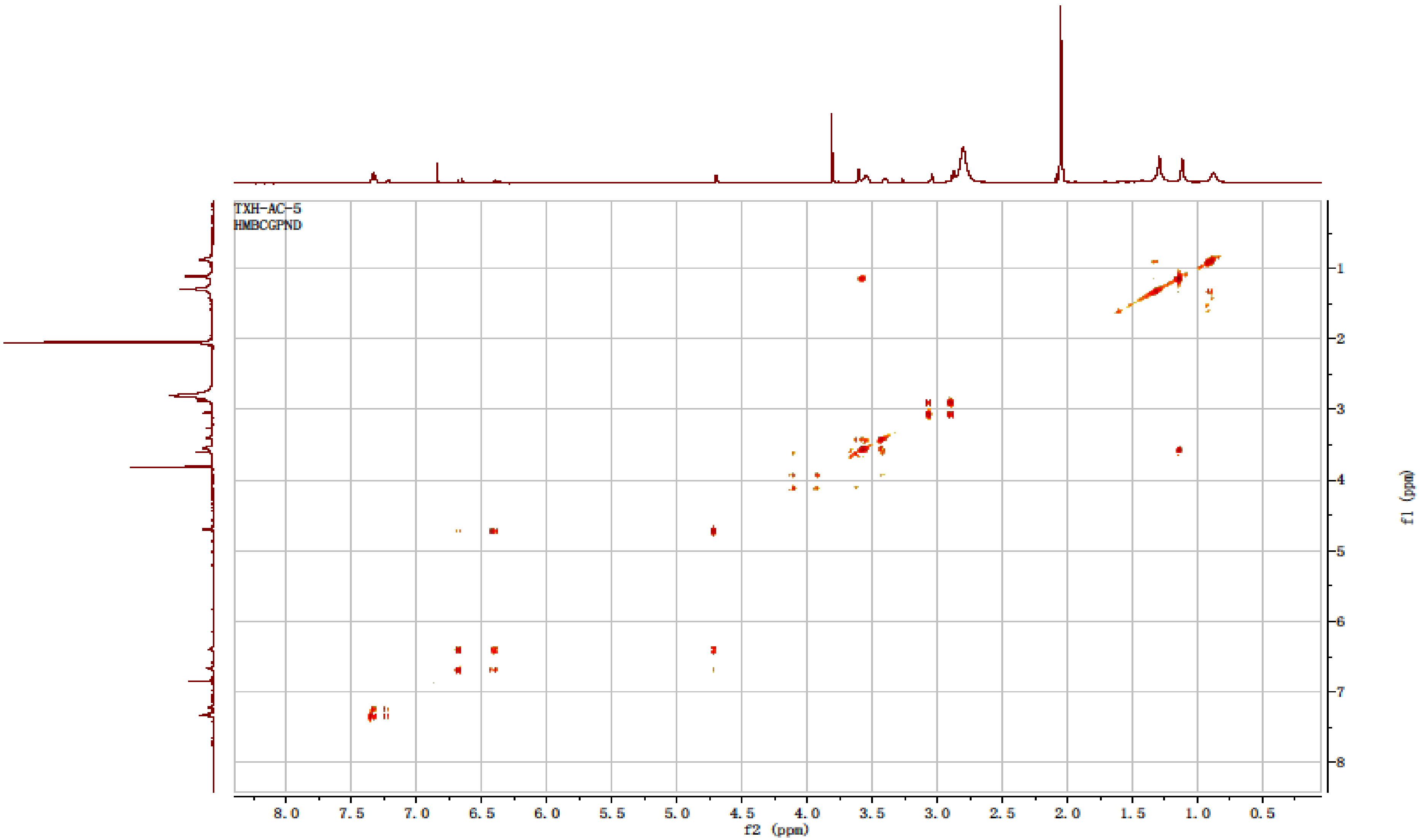

HSQ

Figure S30. C Spectrum of Dichrocephol **D** (**4**; 500 MHz, CD_3COCD_3).

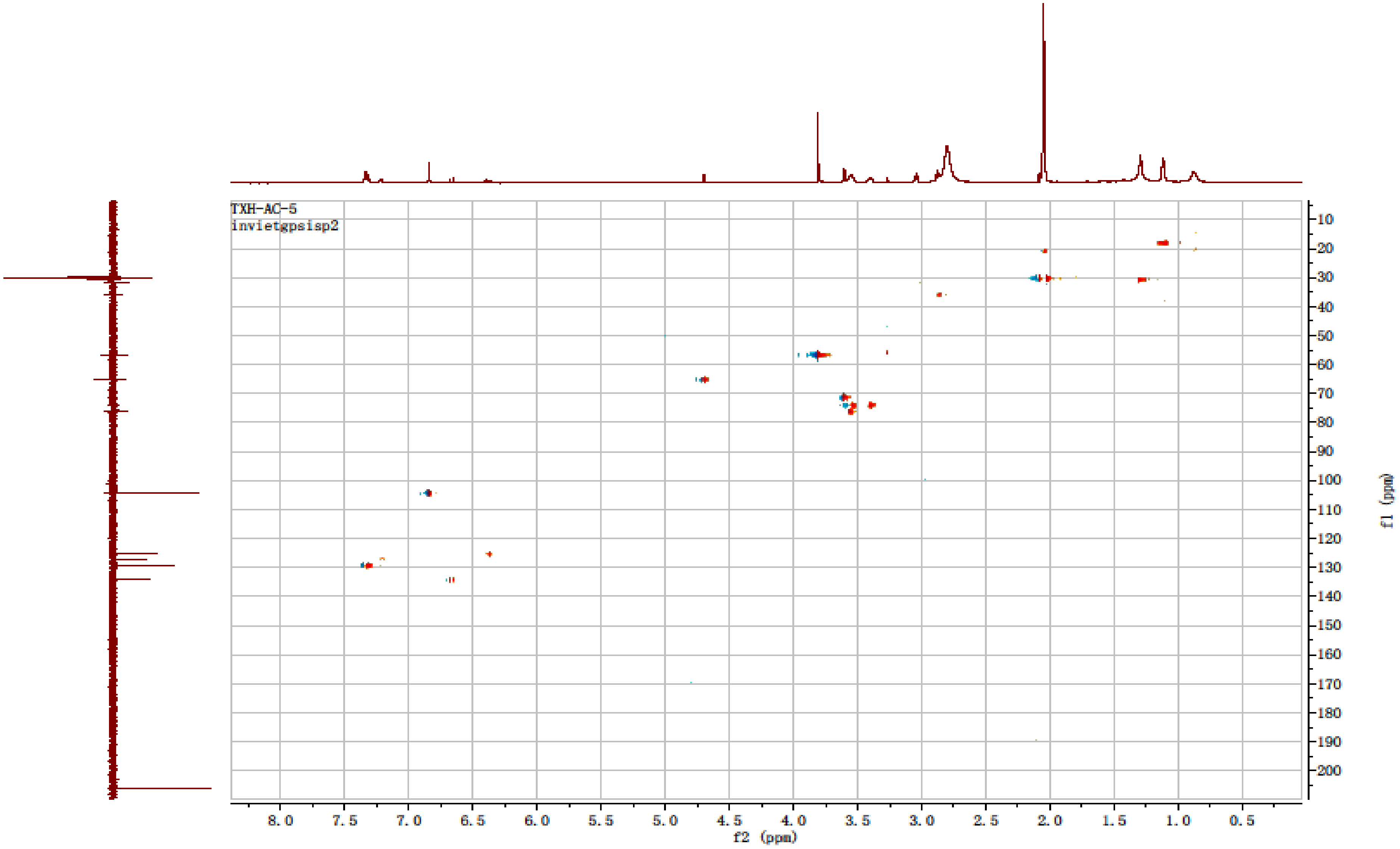

Figure S31. HMBC Spectrum of Dichrocephol **D** (**4**; 500 MHz, CD_3COCD_3).

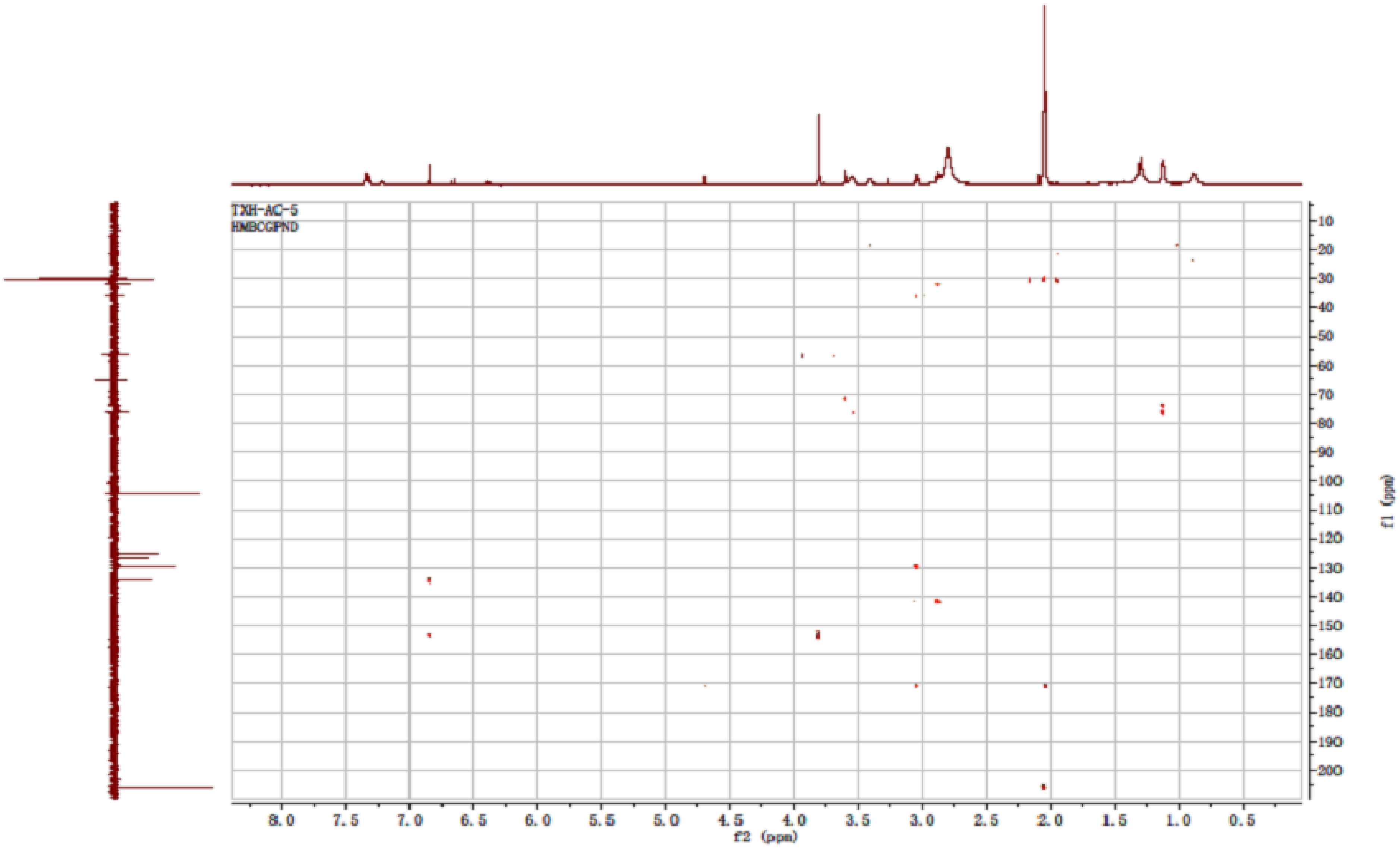

Supplement: Supplementary file 1 [file molecules-18-01720-s001.pdf]
